# Microwave assisted, sequential two-step, one-pot synthesis of novel imidazo[1,2-a]pyrimidine containing tri/tetrasubstituted imidazole derivatives

**DOI:** 10.3906/kim-2009-40

**Published:** 2021-02-17

**Authors:** Tuğba GÜNGÖR

**Affiliations:** 1 Department of Chemistry, Faculty of Sciences and Arts, Natural Products and Drug Research Laboratory,Çanakkale Onsekiz Mart University, Çanakkale Turkey

**Keywords:** Imidazo[1, 2- *a* ]pyrimidine, tri/tetrasubstituted imidazole, microwave synthesis, sequential, one-pot reaction, *p* -toluenesulfonic acid

## Abstract

A series of novel imidazo[1,2- *a* ]pyrimidine containing tri/tetrasubstituted imidazole derivatives (1-10) has been synthesized via sequential two-step, one-pot, multicomponent reaction using imidazo[1,2- *a* ]pyrimidine-2-carbaldehyde, benzil, primary amines, and ammonium acetate catalyzed by *p* -toluenesulfonic acid under microwave-assisted conditions. The results showed that target compounds can be obtained from a wide range of primary amines bearing different functional groups with moderate to good yields (46%-80%) under optimum reaction conditions. This method provides a green protocol for imidazo[1,2- *a* ]pyrimidine containing tri/tetrasubstituted imidazole derivatives due to ethyl alcohol as a green solvent, microwave irradiation as a greener heating method and one-pot multicomponent reaction as a green technique. The synthesized compounds have been elucidated using various spectroscopic tools such as FT-IR, ^1^H NMR, ^13 ^C NMR, and MS.

## 1. Introduction

**Figure 1:**
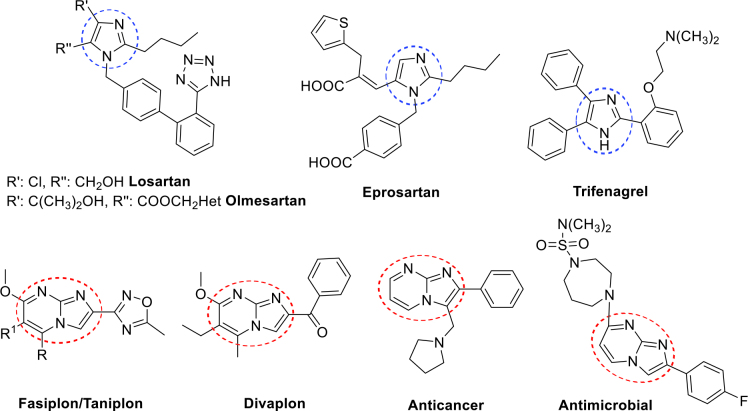
Structures of some biologically active imidazoles and imidazo[1,2-a]pyrimidines.

Nitrogen containing heterocyclic compounds have a great interest within the field of pharmaceutical chemistry and drug industry due to their strong and selective hydrogen bonds with protein/enzyme moieties, which are responsible for important biological activities [1-3]. Imidazole moieties are privileged structures in today’s medicinal chemistry. Also, multisubstituted imidazoles exhibit good pharmaceutical properties such as antibacterial [4], antioxidant [5], anticancer [3], antifungal [5], p38α MAP kinase inhibitor [6], B-Raf kinase inhibitor [7] etc. [8-11]. In addition, imidazole derivatives are used as ionic liquids which are nonvolatile and clean solvents in green chemistry, and materials for energy-based areas [12]. Some APIs such as losartan, eprosartan, and olmesartan are well-known substituted imidazoles, which are used, in the treatment of especially u21b4 (hypertension), indirectly diabetic kidney disease and heart failure and also trifenagrel drug uses, as arachidonate cyclooxygenase inhibitor [1,13-15] (Fig. 1).

On the other hand, imidazo[1,2- *a* ]pyrimidines are an important fused heterocyclic class, which shows significant biological properties such as antiinflammatory [16], cardiovascular [17], anticancer [18,19], antimicrobial [19], p38 MAP kinase inhibitors [20], HIV-1 inhibitor [21], and so on [22,23]. Some of the most successful imidazo[1,2- *a* ]pyrimidine-containing APIs are fasiplon, taniplon, and divaplon, which show anxiolytic and anticonvulsant effects [24-26] (Fig. 1). Also, our group has previously reported the synthesis and characterization of some imine and pyran derivatives of imidazo[1,2- *a* ]pyrimidine and biological studies on these structures are ongoing [27,28]. Hence, to incorporate these two nitrogen containing heterocycles, imidazole and imidazo[1,2- *a* ]pyrimidine, can contribute to obtaining a new class of compounds that may have good biological and medicinal properties. Some of the reported biologically potent imidazole and imidazo[1,2- *a* ]pyrimidine derivatives were given in Figure 1 [18,19].

Various synthetic methodologies were developed to obtain 2,4,5-trisubstituted and 1,2,4,5-tetrasubstituted imidazole derivatives by numerous scientists. One of the most studied methodology is one-pot multicomponent reactions of 1,2-diketone/α-hydroxyketone, primary amines, benzil, and ammonium acetate in the presence of catalysts of different properties such as acidic/basic/neutral, homogeneous/heterogenous, ionic liquids, or nanoparticles using conventional heating, microwave, or ultrasound energies [8,11,29-31]. FeCl_3_.6H_2_O [32], NaH_2_PO_4_ [33], ZnO [10], trityl chloride [13], MCM-41silica or *p* -TsOH [34], benzotriazole [35], HBF4–SiO_2_ or LiBF_4_ [11], TBABr [36], γ-Fe_2_O_3_@TiO_2_-EG-Cu(II) [37] can be given as efficient catalysts in this area. Also, these targets can be synthesized from various starting materials at different reaction conditions such as one-pot condensation of nitriles, amines and benzoin, *N* -alkylation of trisubstituted imidazoles, cyclocondensation of *N* -alkyl-α-acetamidoketone/alcohol with ammonium acetate/ammonium trifluoroacetate, two-step reactions from alkenes through ketoiodonation/cyclisation, [3+2] or [2+2+1] annulation reactions from 2,3-disubstitutedazirines and imines, reaction of aldehydes with α-amido sulfones and so on [8,11,32,38].

Microwave energy is used extensively to heat or to carry out chemical reactions in a wide range of applications such as organic synthesis, polymer/material sciences, nanotechnological, and biochemical procedures since the first report on microwave assisted organic synthesis by Gedye and Giguere/Majetich in 1986 [39-42]. This environmentally friendly technique is applied to a wide variety of reaction types due to its superior properties such as short reaction time, high product yield, fewer by-products, high purity compared with conventional heating [39,40,43]. Also, there are some examples on microwave-promoted synthesis of imine functional group containing heterocyclic compounds and tri/tetrasubstituted imidazole derivatives with one-pot multicomponent reaction in the literature [27,30,44-47]. In addition, one-pot multicomponent approach is a very useful tool to construct the complex compounds in recent years due to the advantages of short reaction time, high atom economy, minimum energy consumption, safety, cheapness, easy applicability, and environmentalist [48].

**Scheme 1:**
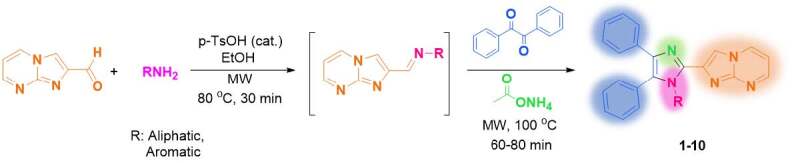
Synthesis of imidazo[1,2-a]pyrimidine containing tri/tetrasubstituted imidazoles.

In this study, one-pot, sequential two step synthesis of imidazo[1,2- *a* ]pyrimidine containing tri and tetrasubstituted novel imidazoles (1-10) from imidazo[1,2- *a* ]pyrimidine-2-carbaldehyde, aliphatic/aromatic amines, benzil, and ammonium acetate in the presence of *p* -TsOH catalyst applying microwave energy was reported for the first time (Scheme 1). Spectroscopic characterizations of products were carried out with FT-IR, ^1^H NMR, ^13^C NMR, and MS analyses. The synthesized compounds are expected to exhibit good biological properties due to having two nitrogen-containing heterocycles, imidazole, and imidazo[1,2- *a* ]pyrimidine as potential pharmacophores.

## 2. Materials and methods

All commercial reagents and solvents were used directly without extra purification. Imidazo[1,2- *a* ]pyrimidine-2-carbaldehyde was prepared according to the literature procedure [49]. Experiments were conducted using by CEM SP Discover microwave synthesis reactor. Thin-layer chromatography was performed to track the reactions on Merck Kieselgel 60GF_254_ aluminum plates using 1:2 or 1:6 hexane:ethyl acetate as mobile phases. Visualization was done by UV at 254 and 366 nm. Chromatographic separations of products were performed on a silica gel column by using Merck silica gel 60, 230 **-** 400 mesh. Product yields were reported after purification steps, unless otherwise stated. All melting points were determined by X-4 melting-point apparatus. The IR spectra of compounds were recorded on a Perkin Elmer Spectrum 100 FTIR spectrophotometer (Waltham, MA USA) by ATR technique and were expressed in cm **-** 1. NMR spectra were recorded in DMSO-d_6_ on Jeol (400 MHz for ^1^H NMR, 100 MHz for ^13^C NMR), and Agilent (600 MHz for ^1^H NMR, 150 MHz for ^13^C NMR) high-performance digital FT-NMR. The chemical shifts (δ) were expressed in ppm and following splitting patterns were used: s, singlet; d, doublet; t, triplet; q, quartlet; m, multiplet and dd, double doublet. LC-MS analyses were performed with Shimadzu LC-MS/MS 8040 liquid chromatograph mass spectrometer equipped with an electrospray ionization source. Molecular weights of products were determined in positive ion mode monitoring [M+H]^+^ species. Compounds were named following IUPAC rules via ChemDraw 16.0 program (PerkinElmer, Inc., Waltham, MA USA).

**General procedure for the preparation of tri/tetrasubstituted imidazo[1,2-** a **]pyrimidine (1-10)**

A mixture of imidazo[1,2- *a* ]pyrimidine-2-carbaldehyde [49] (75 mg, 0.51 mmol), amine derivative (0.56 mmol, 1.1 equiv.), and *p* -toluenesulfonic acid (cat., 20% mol, 19.4 mg) was suspended in ethyl alcohol (2 mL) in a 35 mL microwave reaction vessel and stirred at room temperature for 5 min. The mixture was heated at 80 °C for 30 min by applying 100 W microwave energy. The mixture was cooled down to room temperature, benzil (0.107 g, 0.51 mmol, 1.0 equiv.), and ammonium acetate (0.196 g, 2.55 mmol, 5.0 equiv.) were added to the reaction medium and stirred at room temperature for 5 min. Microwave irradiation (200 W) was applied to the mixture at 100 °C for 60-80 min until the reaction completion (TLC monitoring with hexane/ethyl acetate 1:2 and 1:6). The mixture was cooled to room temperature, and solvent/volatiles were evaporated under vacuum. DCM (20 mL) was added to the residue, and organic phase was washed with distilled water (20 mL). Organic layer was dried over Na_2_SO_4_, filtered, and evaporated. The crude product was purified by column chromatography using hexane:ethyl acetate 1:4 or 1:6 as eluent. In case of minor impurities, the residue was treated with Et_2_O to obtain pure products after chromatographic purification. The following compounds (1-10) were prepared according to this general method.

**2-(4,5-Diphenyl-1H-imidazol-2-yl)imidazo[1,2-a]pyrimidine (1)**

The crude product which was obtained with one-step microwave reaction of aldehyde, benzil, ammonium acetate, and *p* -TsOH at 100 °C was purified by column chromatography using ethyl acetate. Yellow solid; yield: 100 mg (58%); m.p.: 290-292 °C (dec.); R_f_= 0.40 (ethyl acetate); ^1^H NMR (600 MHz, DMSO-d_6_): δ = 13.20 (s, 1H, -NH), 9.01 (dd, 1H, *J* = 6.7, 1.9 Hz), 8.56 (dd, 1H, *J* = 4.2, 1.9 Hz), 8.36 (s, 1H), 7.51-7.47 (m, 4H), 7.37 (t, 2H, *J* = 7.6 Hz), 7.32-7.29 (m, 3H), 7.22 (t, 1H, *J* = 7.6 Hz), 7.11 (dd, 1H, *J* = 6.7, 4.2 Hz) ppm; ^13^C NMR (150 MHz, DMSO-d_6_): δ = 151.1, 148.2, 142.1, 138.3, 138.1, 135.7, 131.2, 129.3, 128.9, 128.8, 128.6, 128.5, 128.0, 127.8, 127.1, 109.7, 109.1 ppm; IR (ATR): ϑ= 3141, 3062, 3026, 1618, 1603, 1524, 1505, 1489, 1445, 1419, 1342, 1311, 1244, 1229, 1109, 1072, 969, 917, 763, 693 cm^-1^; MS: m/z (%) = 338 (M^+^, 100), 327 (44), 301 (64).

**2-(1,4,5-Triphenyl-1H-imidazol-2-yl)imidazo[1,2-a]pyrimidine (2)**

Yellow-green solid; yield: 105 mg (50%); m.p.: 234-235 °C; R_f_= 0.40 (hexane:ethyl acetate, 1:6); ^1^H NMR (400 MHz, DMSO-d_6_): δ = 10.20 (dd, 1H, *J* = 7.0, 2.1 Hz), 8.66 (dd, 1H, *J* = 4.1, 2.1 Hz), 7.56 (d, 2H, *J* = 7.3 Hz), 7.46 (s, 5H), 7.34 (dd, 1H, *J* = 7.0, 4.1 Hz), 7.30 (s, 5H), 7.26 (d, 2H, *J* = 7.7 Hz), 7.20 (d, 1H, *J* = 7.3 Hz), 6.55 (s, 1H) ppm; ^13^C NMR (100 MHz, DMSO-d_6_): δ = 151.6, 148.7, 138.4, 137.3, 136.5, 136.3, 134.5, 134.1, 131.6, 131.2, 130.33, 130.26, 130.2, 129.6, 129.2, 129.1, 128.9, 127.3, 126.9, 114.4, 110.6 ppm; IR (ATR): ϑ= 3093, 3058, 3033, 3009, 1615, 1594, 1580, 1516, 1492, 1417, 1401, 1367, 1282, 1251, 1160, 1093, 1071, 942, 860, 794, 768, 692, 657 cm^-1^; MS: m/z (%) = 414 (M+, 100), 391 (44), 381 (6).

**2-(4,5-Diphenyl-1-p-tolyl-1H-imidazol-2-yl)imidazo[1,2-a]pyrimidine (3)**

Yellow-green solid; yield: 131 mg (60%); m.p.: 233 °C; Rf= 0.45 (hexane:ethyl acetate, 1:6); ^1^H NMR (600 MHz, DMSO-d_6_): δ = 10.23 (dd, 1H, *J* = 7.0, 1.5 Hz), 8.67-8.66 (m, 1H), 7.56 (d, 2H, *J* = 7.6 Hz), 7.35-7.26 (m, 12H), 7.20 (t, 1H, *J* = 7.0 Hz), 6.57 (s, 1H), 2.34 (s, 3H) ppm; ^13^C NMR (150 MHz, DMSO-d_6_): δ = 151.5, 148.6, 139.9, 138.4, 137.1, 136.2, 134.4, 134.0, 133.8, 131.5, 131.1, 130.7, 130.2, 129.1, 129.0, 128.8, 127.2, 126.7, 114.3, 110.5, 21.2 ppm; IR (ATR): ϑ= 3093, 3056, 3047, 3034, 3007, 2960, 2927, 1617, 1601, 1579, 1516, 1496, 1429, 1421, 1370, 1287, 1243, 1161, 1129, 1111, 1019, 941, 914, 830, 798, 783, 765, 693, 656 cm^-1^; MS: m/z (%) = 428 (M^+^, 100), 413 (12), 405 (8), 392 (28), 381 (6).

**4-(2-(Imidazo[1,2-a]pyrimidin-2-yl)-4,5-diphenyl-1H-imidazol-1-yl)phenol (4)**

Cream-colored solid; yield: 123 mg (56%); m.p.: >300 °C; R_f_= 0.28 (hexane:ethyl acetate, 1:6); ^1^H NMR (600 MHz, DMSO-d_6_): δ = 10.26 (dd, 1H, *J* = 7.1, 1.9 Hz), 9.99 (s, 1H, -OH), 8.67 (dd, 1H, *J* = 4.0, 1.9 Hz), 7.56 (d, 2H, *J* = 8.1 Hz), 7.35-7.32 (m, 4H), 7.30-7.23 (m, 6H), 7.19 (t, 1H, *J* = 7.1 Hz), 6.80 (d, 2H, *J* = 8.1 Hz), 6.63 (s, 1H) ppm; ^13^C NMR (150 MHz, DMSO-d_6_): δ = 158.7, 151.4, 148.6, 138.7, 136.9, 136.2, 134.5, 134.0, 131.5, 131.3, 130.5, 130.3, 129.1, 129.0, 128.7, 127.3, 127.1, 126.7, 116.5, 114.4, 110.5 ppm; IR (ATR): ϑ= 3092, 3066, 3013, 1622, 1603, 1516, 1503, 1479, 1446, 1407, 1373, 1279, 1240, 1165, 1133, 1099, 1044, 945, 840, 788, 770, 733, 702, 692, 662 cm^-1^; MS: m/z (%) = 430 (M+, 100), 413 (20), 391 (36), 327 (20), 301 (16).

**2-(1-(4-Methoxyphenyl)-4,5-diphenyl-1H-imidazol-2-yl)imidazo[1,2-a]pyrimidine (5)**

Yellow solid; yield: 140 mg (62%); m.p.: 236-238 °C; Rf = 0.38 (hexane:ethyl acetate, 1:6); ^1^H NMR (600 MHz, DMSO-d_6_): δ = 10.25 (dd, 1H, *J* = 7.1, 1.6 Hz), 8.67 (dd, 1H, *J* = 3.9, 1.6 Hz), 7.56 (d, 2H, *J* = 7.7 Hz), 7.40 (d, 2H, *J* = 8.6 Hz), 7.36-7.32 (m, 6H), 7.28 (t, 2H, *J* = 7.7 Hz), 7.20 (t, 1H, *J* = 7.1 Hz), 6.99 (d, 2H, *J* = 8.6 Hz), 6.60 (s, 1H), 3.77 (s, 3H) ppm; ^13^C NMR (150 MHz, DMSO-d_6_): δ = 160.2, 151.5, 148.6, 138.7, 137.0, 136.2, 134.4, 134.0, 131.5, 131.3, 130.6, 130.3, 129.1, 129.0, 128.8, 128.7, 127.1, 126.7, 115.2, 114.4, 110.5, 55.9 ppm; IR (ATR): ϑ= 3106, 3065, 3018, 2947, 2923, 2851, 1615, 1603, 1580, 1513, 1496, 1444, 1404, 1367, 1283, 1252, 1165, 1107, 1015, 941, 836, 786, 768, 714, 694, 657 cm^-1^; MS: m/z (%) = 444 (M^+^, 100), 413 (4), 391 (16), 329 (10), 310 (12).

**2-(1-(4-Chlorophenyl)-4,5-diphenyl-1H-imidazol-2-yl)imidazo[1,2-a]pyrimidine (6)**

Yellow solid; yield: 105 mg (46%); m.p.: 255-256 °C; Rf= 0.53 (hexane:ethyl acetate, 1:6); ^1^H NMR (600 MHz, DMSO-d_6_): δ = 10.15 (dd, 1H, *J* = 7.1, 1.9 Hz), 8.68 (dd, 1H, *J* = 4.0, 1.9 Hz), 7.57 (d, 2H, *J* = 7.7 Hz), 7.53 (s, 4H), 7.36-7.34 (m, 4H), 7.32-7.30 (m, 2H), 7.28 (d, 2H, *J* = 7.7 Hz), 7.21 (t, 1H, *J* = 7.1 Hz), 6.70 (s, 1H) ppm; ^13^C NMR (150 MHz, DMSO-d_6_): δ = 151.7, 148.7, 138.2, 137.2, 136.1, 135.3, 134.8, 134.2, 134.1, 131.6, 131.4, 131.1, 130.2, 129.9, 129.3, 129.2, 128.8, 127.3, 126.7, 114.2, 110.6 ppm; IR (ATR): ϑ= 3095, 3069, 3058, 3049, 3008, 1616, 1601, 1520, 1493, 1476, 1403, 1370, 1287, 1249, 1161, 1129, 1088, 1015, 941, 842, 803, 782, 766, 694, 654 cm^-1^; MS: m/z (%) = 448 (M^+^, 100), 428 (6), 413 (8), 391 (36).

**2-(1-Benzyl-4,5-diphenyl-1H-imidazol-2-yl)imidazo[1,2-a]pyrimidine (7)**

Cream-colored solid; yield: 148 mg (68%); m.p.: 245-246 °C; Rf= 0.30 (hexane:ethyl acetate, 1:6); ^1^H NMR (600 MHz, DMSO-d_6_): δ = 9.01 (dd, 1H, *J* = 6.7, 1.9 Hz), 8.54 (dd, 1H, *J* = 4.0, 1.9 Hz), 8.49 (s, 1H), 7.44-7.39 (m, 5H), 7.22-7.19 (m, 4H), 7.15-7.10 (m, 5H), 6.79 (d, 2H, *J* =7.3 Hz), 5.88 (s, 2H, benzyl -CH2) ppm; ^13^C NMR (100 MHz, DMSO-d_6_): δ = 151.3, 147.8, 140.9, 138.5, 138.4, 137.8, 135.6, 134.7, 131.3, 131.0, 130.6, 129.4, 129.3, 128.8, 128.7, 128.6, 127.7, 127.4, 126.9, 126.6, 111.4, 110.0, 48.0 ppm; IR (ATR): ϑ= 3121, 3096, 3063, 3028, 2938, 1618, 1602, 1583, 1506, 1496, 1434, 1400, 1330, 1309, 1232, 1215, 1150, 1074, 1027, 940, 921, 787, 779, 757, 723, 691 cm^-1^; MS: m/z (%) = 428 (M^+^, 100), 413 (36), 391 (44), 327 (28), 310 (20).

**2-(1-Ethyl-4,5-diphenyl-1H-imidazol-2-yl)imidazo[1,2-a]pyrimidine (8)**

Cream-colored solid; yield: 149 mg (80%); m.p.: 180-183 °C; Rf= 0.38 (hexane:ethyl acetate, 1:8); ^1^H NMR (400 MHz, DMSO-d_6_): δ = 9.03 (dd, 1H, *J* = 6.7, 1.9 Hz), 8.57 (dd, 1H, *J* = 4.0, 1.9 Hz), 8.46 (s, 1H), 7.57-7.52 (m, 3H), 7.47-7.46 (m, 2H), 7.40 (d, 2H, *J* = 7.7 Hz), 7.19 (t, 2H, *J* = 7.7 Hz), 7.14-7.10 (m, 2H), 4.46 (q, 2H, *J* = 7.0 Hz), 1.14 (t, 3H, *J* = 7.0 Hz) ppm; ^13^C NMR (150 MHz, DMSO-d_6_): δ = 151.1, 148.0, 140.1, 138.7, 137.4, 135.5, 134.9, 131.5, 131.1, 130.6, 129.7, 129.5, 128.5, 126.6, 126.4, 111.1, 109.9, 39.9, 16.8 ppm; IR (ATR): ϑ= 3102, 3083, 3060, 3023, 2979, 2955, 2935, 1619, 1601, 1576, 1525, 1504, 1443, 1376, 1305, 1247, 1233, 1174, 1130, 1071, 934, 919, 790, 765, 690 cm^-1^; MS: m/z (%) = 366 (M^+^, 100), 338 (10), 327 (14), 310 (14).

**2-(1-Isopropyl-4,5-diphenyl-1H-imidazol-2-yl)imidazo[1,2-a] pyrimidine (9)**

White solid; yield: 139 mg (72%); m.p.: 278-279 °C; Rf= 0.35 (hexane:ethyl acetate, 1:8); ^1^H NMR (600 MHz, DMSO-d_6_): δ = 9.03 (d, 1H, *J* = 6.5 Hz), 8.58 (s, broad, 1H), 8.41 (s, 1H), 7.54 (s, broad, 3H), 7.49 (s, broad, 2H), 7.32 (d, 2H, *J* = 7.5 Hz), 7.17-7.13 (m, 3H), 7.09 (t, 1H, *J* = 6.5 Hz), 5.31 (s, broad, 1H), 1.38 (d, 6H, *J* = 6.7 Hz) ppm; ^13^C NMR (150 MHz, DMSO-d_6_): δ = 151.2, 147,6, 140.5, 139.0, 137.6, 135.5, 135.0, 132.4, 132.3, 130.6, 129.6, 129.4, 128.4, 126.5, 126.4, 112.2, 109.9, 49.4, 22.8 ppm; IR (ATR): ϑ= 3116, 3092, 3056, 2973, 2934, 2875, 1651, 1622, 1603, 1575, 1523, 1504, 1442, 1360, 1304, 1274, 1239, 1179, 1148, 1072, 939, 917, 778, 763, 697, 653 cm^-1^; MS: m/z (%) = 380 (M^+^, 100), 338 (12), 327 (16), 310 (12).

**2-(1-Cyclohexyl-4,5-diphenyl-1H-imidazol-2-yl)imidazo[1,2-a]pyrimidine (10)**

White solid; yield: 145 mg (68%); m.p.: 267-268 °C; R_f_= 0.25 (hexane:ethyl acetate, 1:6); ^1^H NMR (600 MHz, DMSO-d_6_): δ = 9.03 (dd, 1H, *J* = 7.0, 2.0 Hz), 8.58 (dd, 1H, *J* = 4.1, 2.0 Hz), 8.40 (s, 1H), 7.55-7.54 (m, 3H), 7.47-7.46 (m, 2H), 7.32 (d, 2H, *J* = 7.4 Hz), 7.17-7.13 (m, 3H), 7.09 (t, 1H, *J* = 7.0 Hz), 1.91-1.83 (m, 5H), 1.65 (d, 2H, *J* = 13.1 Hz), 1.48 (d, 1H, *J* = 13.1 Hz), 1.12 (q, 2H, *J* = 13.1 Hz), 0.85 (m, 1H) ppm; ^13^C NMR (150 MHz, DMSO-d_6_): δ = 151.2, 147.6, 140.7, 139.0, 135.5, 135.0, 132.2, 130.7, 129.7, 129.3, 128.4, 126.5, 126.4, 112.2, 109.9, 57.7, 32.5, 26.3, 25.1 ppm; IR (ATR): ϑ= 3102, 3080, 3063, 3026, 2945, 2923, 2856, 1619, 1603, 1575, 1525, 1505, 1442, 1391, 1288, 1277, 1235, 1070, 1007, 936, 916, 892, 785, 767, 696, 671 cm^-1^; MS: m/z (%) = 420 (M+, 100), 391 (28), 338 (8), 310 (12).

## 3. Results and Discussion

In the present work, a new series of 2-(1-substituted-4,5-diphenyl-1 *H* -imidazol-2-yl)imidazo[1,2- *a* ]pyrimidine (1–10) is reported (Scheme 1). Firstly, the synthesis of 2-(4,5-diphenyl-1- *p* -tolyl-1 *H* -imidazol-2-yl)imidazo[1,2- *a* ]pyrimidine **(3)** from imidazo[1,2- *a* ]pyrimidine-2-carbaldehyde, *p* -toluidine, benzil, and ammonium acetate was determined as a model reaction, and some optimization studies under microwave irradiation were carried out on it (Table 1).

**Table 1:** Various reaction conditions for the model compound 3.

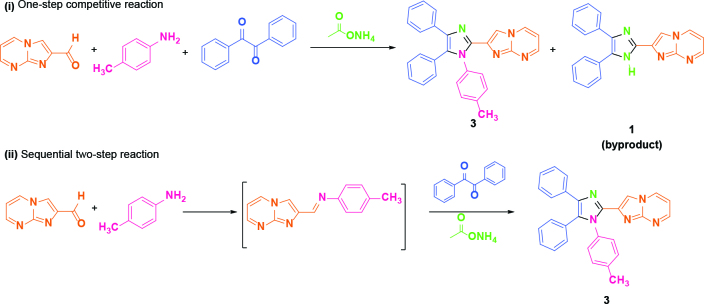						
Entry	Solvent	Amount of NH4OAc	Catalyst/Amount	Temperature (°C) /Power (Watt)	Time (min)	Yielda (%)
1	EtOH	1.0 equiv.	-	100/100	90	20b
2	AcOH	1.0 equiv.	AcOH	140/200	30	25b
3	EtOH	1.0 equiv.	FeCl_3_.6H_2_O/5% mol	120/200	45	28b
4	-	1.0 equiv.	NaH_2_PO_4_/30% mol	120/100	30	30b
5	EtOH	1.0 equiv.	Trityl chloride/ 10% mol	120/200	45	30b
6	EtOH	1.0 equiv.	p-TsOH/20% mol	120/200	40	36b
7	EtOH	5.0 equiv.	p-TsOH/20% mol	80/100 100/200	3080	60c
8	1-butanol	3.0 equiv.	p-TsOH/20% mol	80/100 120/200	30 30	44c
9	Toluene	5.0 equiv.	AcOH/5 equiv.	80/100120/200	30 30	42c
10	-	5.0 equiv.	p-TsOH/20% mol	80/100 100/200	3030	40c

a Isolated yield. b One-step reaction. c Sequential two-step reaction.

Firstly, one-pot, one-step, multicomponent reaction conditions were applied. It was observed that the conversion rate was approximately 50% at 90 min reaction time and the isolated product yield was 20% with no catalyst in ethyl alcohol in 100 °C temperature (Table 1, entry 1). During optimization studies, AcOH, FeCl_3_.6H_2_O, NaH_2_PO_4_, trityl chloride and *p* -TsOH were used to explore the impact of different catalysts (Table 1, entry 2-6). Multicomponent reaction in acetic acid at 140 °C resulted in 25% product yield (Table 1, entry 2). In the presence of acetic acid, *N* -( *p* -tolyl)acetamide was isolated as a by-product which obtained by amidation reaction between acetic acid and *p* -toluidine. It was determined that almost all catalysts work in our model reaction, but the best catalyst is *p* -TsOH in terms of product yield.

Consequently, the preliminary one-pot, one-step results showed us reactions were carried out in a competitive way and a certain amount of 2,4,5-trisubstituted imidazole **(1)** was also obtained in addition to main tetrasubstituted product although different catalysts were used [11]. Therefore, next efforts were directed to the one-pot, sequential two step reactions to prevent trisubstituted imidazole by-product. For this purpose, the imine formation reaction was carried out between aldehyde, primary amine, and catalyst in the first step and then benzil and ammonium acetate were added to the reaction medium to conduct condensation and cyclization in the second step.

While the usage of 5.0 equiv. ammonium acetate cause to increasement of product yield in two-step reactions, it was used 1.0 equiv. in one-step multicomponent reaction to minimize the formation of trisubstituted imidazole. While the reaction was carried out neat conditions or higher temperatures such as 120 °C in different solvent choice, the starting materials (aldehyde or imine) consumed in shorter reaction times, but the ratio of decomposition by-product increased and the yields of target product decreased. Also, the use of more *p* -TsOH as catalyst did not lead to any increase in imidazole formation.

When the reaction was run in the sequential two-step with the catalyst of *p* -TsOH under microwave irradiation at 80 °C for 30 min and 100 °C for 80 min, the best result was obtained for compound 3 (Table 1, entry 7). Synthetic details were reported in the experimental section.

Followed by the primary results, this method was applied to the various imidazo[1,2- *a* ]pyrimidine containing tri/tetrasubstituted imidazole derivatives using different aliphatic (ethyl, isopropyl, cyclohexyl and benzyl amines) and electron donating or withdrawing functional groups bearing aromatic primary amines (aniline, *p* -toluidine, *p* -aminophenol, *p* -anisidine, *p* -chloroaniline) at optimized reaction conditions (Table 2). Also, 2,4,5-trisubstituted imidazole derivative (1) was synthesized successfully (58%) according to the specified reaction conditions. While aromatic derivatives were obtained in 46%-62% yields, aliphatic derivatives were synthesized in higher yields (68%-80%). In addition, electron withdrawing group (-Cl) bearing compound (6) was obtained with lower yield compared to the other aromatic derivatives. Interestingly, the reaction of aldehyde and 3-amino-5-methylisoxazole as a heterocyclic primary amine to obtain their Schiff base was found unsuccessful in these conditions, so heterocyclic unit containing target compound was not synthesized.

**Table 2:** Synthesis of tri/tetrasubstituted imidazoles (1-10).

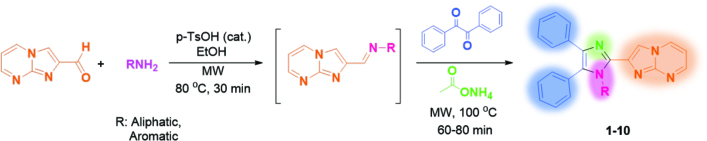			
Entry	Amine	Producta	Yieldb (%)
1	-	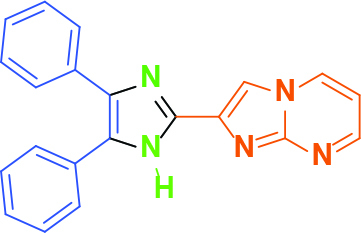	58
2	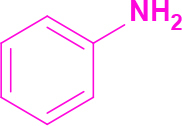	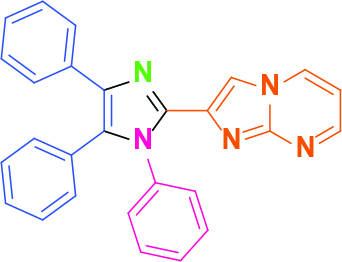	50
3	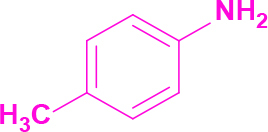	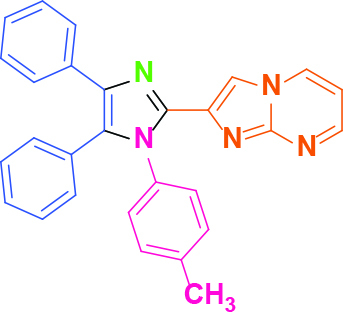	60
4	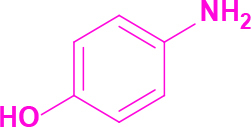	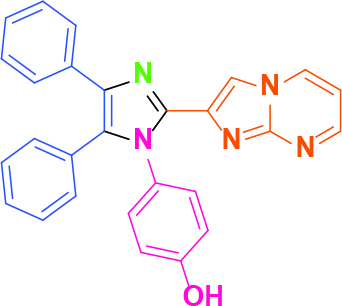	56
5	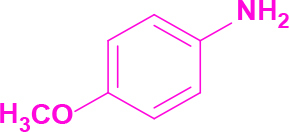	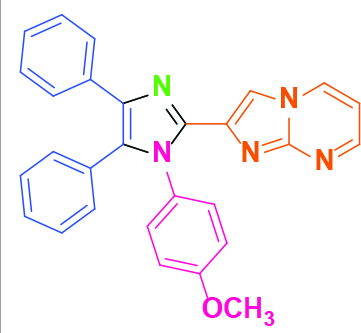	62
6	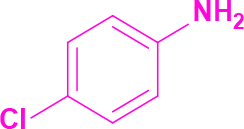	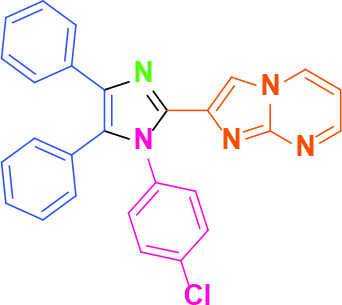	46
7	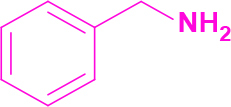	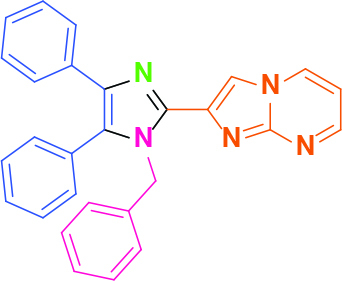	68
8	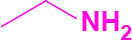	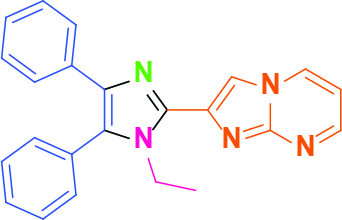	80
9	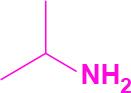	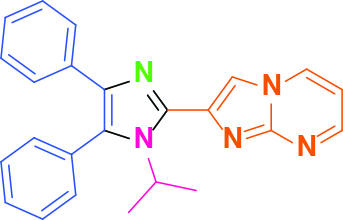	72
10	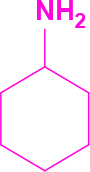	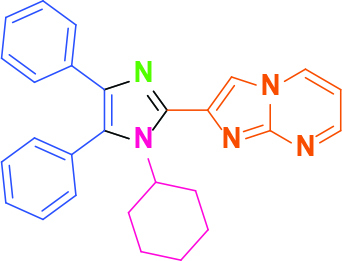	68

a Reaction conditions: Imidazo[1,2-a]pyrimidine-2-carbaldehyde (1.0 equiv.), amine (1.1 equiv.), benzil (1.0 equiv.), ammonium acetate (5.0 equiv.), catalyst (20%). b Yields refer to the isolated pure products.

The optimized condition (Table 1, entry 7) for compound 3 was adapted to the conventional method as reflux in ethyl alcohol. While the first step resulted in successful imine formation in 5 h, the final imidazole product was obtained at long reaction times (36 h) with low yields (30%) in the second step compared to microwave outputs. Also, another conventional reaction was carried out in the absence of *p* -TsOH catalyst and the conversion of imine to imidazole was not observed at 24 h reflux in EtOH. As a result, the multidimensional positive effects (high yield, short reaction times, and low by-product formation) of the catalyst and microwave energy on the reaction were clearly observed.

**Figure 2:**
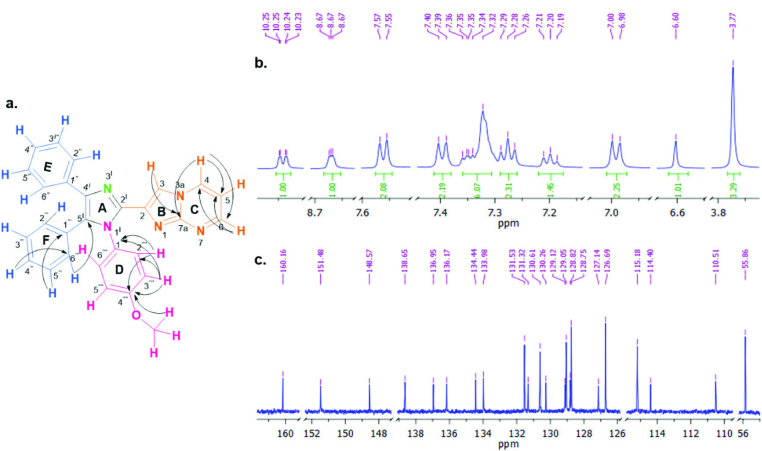
a. Important HMBC correlations. b. ^1^H NMR spectra. c. ^13^C NMR spectra of compound 5.

All the imidazole derivatives were confirmed by using MS, FT-IR, 1H, and ^13^C NMR spectral characterization and melting points (see experimental and supporting information part). According to the FT-IR spectra, the absence of C=O stretching of benzil and aldehyde derivative, -NH2 stretching of amines and the presence of aromatic C-H stretching in 3000-3100 cm^-1^, C=C and C=N absorptions in 1625-1575 cm^-1^ and strong out-of-plane C-H bending vibrations of mono/disubstituted benzenes in 900-650 cm^-1^ confirm the structure of final products. Taking compound 5 as an example, the structure was confirmed spectroscopically with 1H and ^13^C NMR and also 2D NMR analysis (HSQC and HMBC) (see supporting information). Chemical shifts and *J* couplings of the ^1^H NMR spectrum showed the presence of two doublet of doublets at 10.25, 8.67 and singlet peak at 6.60 ppm corresponding to protons H-4, H-6 of ring C and proton H-3 of ring B, respectively and singlet peak at 3.77 ppm corresponding to the methoxy group. Other hydrogen signals in the range of 7.57-6.98 ppm belong to the three phenyl rings and H-5. It is observed that doublet peaks at 7.40 and 6.99 ppm with *J* *H-H* = 8.6 Hz corresponded to H-2´´´´ and H-3´´´´ at ring D with AA’BB’ system like as other para-substituted benzene derivatives (3, 4 and 6). There are 1 aliphatic (-OCH3) and 21 aromatic peaks six of which have 2C integration (symmetrical carbons) in ^13^C NMR spectra. According to HSQC interactions, it was concluded that signals at 151.48, 136.12, 133.98, 130.61, 127.14, 126.69, 115.18, and 55.76 ppm corresponded to carbons C-6, C-4, C-3, C-2´´´´/C-6´´´´, C-4´´´, C-2´´´/C-6´´´, C-3´´´´/C-5´´´´ and -OCH3, respectively. Chemical shifts of carbons C-4´´´´ (160.13 ppm, highest value) and C-1´´´´ (128.82 ppm) at ring D were detected as a result of the HMBC correlations with protons H-2´´´´, H-3´´´´, -OCH3 and protons H-2´´´´, H-3´´´´, respectively. While the signal of 110.51 ppm corresponds to the C-5 at ring C due to the HMBC correlations with H-4 and H-6 protons, 148.57 ppm can be the joint carbon (C-7a) of ring B and C due to the strong HMBC interactions with H-3 and H-4. Also, 110.51 ppm (C-5) gives HSQC correlation with 7.36 ppm/H-5 which is overlapped with the multiplet peaks at 7.36-7.32 ppm with 6H integration. In addition, triplet (1H), triplet (2H), and doublet (2H) peaks at 7.20, 7.28 and 7.56 ppm belong to the protons H-4´´´, H-3´´´/H-5´´´ and H-2´´´/H-6´´´at ring F, respectively according to the *J* *H-H* couplings and HMBC results. Some important HMBC correlations, ^1^H NMR and ^13^C NMR spectra of compound 5 were given in Figure 2. Calculated and measured m/z values of the final compounds (1-10) were also found compatible in LC-MS analysis.

**Scheme 2:**
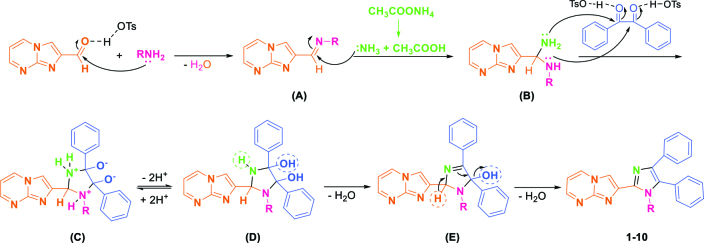
A proposed mechanism for target imidazoles.

A proposed mechanism for imidazo[1,2- *a* ]pyrimidine containing tri/tetrasubstituted imidazoles, which was supported by the literature, was given in Scheme 2 [8,10,13,50]. In the first step, imidazo[1,2- *a* ]pyrimidine-2-carbaldehyde reacts with amine derivative in the presence of catalyst to form imine intermediate (A). Ammonium acetate which was added to the reaction medium in the second step converts to ammonia and acetic acid. Ammonia attacks as a nucleophile to the C=N double bond of the intermediate (A) to produce intermediate (B). The next step consists of the simultaneous double condensation between intermediate (B) and benzil and formation of 5-membered ring (intermediate C). After cyclization, intramolecular proton exchange occurs between nitrogen and oxygen species and forms intermediate (D). Finally, target structures (1-10) are obtained as a result of dehydration step (2 mol H_2_O) with electronic rearrangements. The function of catalyst, *p* -toluenesulfonic acid (TsOH), is to make easy the nucleophilic attack of nitrogen sources (RNH2 and NH3) through the increasement of electrophilic power on carbonyl carbons of aldehyde and benzil starting materials.

In conclusion, microwave-promoted synthesis of novel imidazo[1,2- *a* ]pyrimidine containing tri/tetrasubstituted imidazole derivatives (1-10) via sequential two-step, one-pot, multicomponent reaction of imidazo[1,2- *a* ]pyrimidine-2-carbaldehyde, benzil, primary amines and ammonium acetate using *p* -toluenesulfonic acid as catalyst was described. It was observed that this efficient method was applicable to various primary amines such as aliphatic and aromatic amines bearing electron withdrawing or donating functional groups. Target compounds were obtained with moderate to good yields (46%-80%) under microwave conditions as a green technique. The products were confirmed using several spectroscopic techniques including FT-IR, ^1^H NMR, ^13^C NMR, and MS. Also the structure of compound 5 has been confirmed using HSQC and HMBC as 2D NMR analyses. The biological studies of products (1-10), which are expected to show good pharmacological properties, are ongoing in our research group.

Supplementary Materials

## References

[ref1] (2014). Analysis of the structural diversity, substitution patterns, and frequency of nitrogen heterocycles among U.S. FDA approved pharmaceuticals. Journal of Medicinal Chemistry.

[ref2] (2006). Ytterbium triflate as an efficient catalyst for one-pot synthesis of substituted imidazoles through three-component condensation of benzil, aldehydes and ammonium acetate. Journal of Fluorine Chemistry.

[ref3] (2014). Imidazole derivatives show anticancer potential by inducing apoptosis and cellular senescence. Medicinal Chemistry Communications.

[ref4] (2017). Synthesis and biological evaluation of 1.

[ref5] (2018). The synthesis of imidazoles and evaluation of their antioxidant and antifungal activities. Monatshefte für Chemie-Chemical Monthly.

[ref6] (2008). pyridines as potent p38a MAP kinase inhibitors with excellent in vivo antiinflammatory properties. Imidazolyl benzimidazoles and imidazo[4.

[ref7] (2006). The identification of potent and selective imidazole-based inhibitors of B-Raf kinase. Bioorganic & Medicinal Chemistry Letters.

[ref8] (2019). -tetrasubstituted 1H-imidazole derivatives: State of the art. Catalytic synthesis of 1.

[ref9] (2015). One-pot synthesis of multisubstituted imidazoles under solvent-free conditions and microwave irradiation using Fe3O4@SiO_2_–imid–PMAn magnetic porous nanospheres as a recyclable catalyst. New Journal of Chemistry.

[ref10] (2011). -trisubstituted imidazoles by zinc oxide as efficient and reusable catalyst Monatshefte für Chemie-. synthesis of 1.

[ref11] (2012). Catalytic procedures for multicomponent synthesis of imidazoles: selectivity control during the competitive formation of tri- and tetrasubstituted imidazoles. Green Chemistry.

[ref12] (2009). Designing imidazole-based ionic liquids and ionic liquid monomers for emerging technologies. Polymer Reviews.

[ref13] (2014). -tetrasubstitutedimidazoles catalyzed by trityl chloride in neutral media. One pot synthesis of 1.

[ref14] (1989). Trifenagrel: A chemically novel platelet aggregation inhibitor. Journal of Pharmacology and Experimental Therapeutics.

[ref15] (2019). Urea–zinc chloride eutectic mixture-mediated one-pot synthesis of ımidazoles: efficient and ecofriendly access to trifenagrel. Synlett.

[ref16] (2008). Synthesis and anti-inflammatory activity of imidazo[1,2-a]pyrimidine derivatives. Chinese Chemical Letters.

[ref17] (1988). -a]pyrimidines and imidazo. Journal of Medicinal Chemistry.

[ref18] (2015). Synthesis and antiproliferative activity of imidazo[1,2-a]pyrimidine Mannich bases. European Journal of Medicinal Chemistry.

[ref19] (2019). Rational design, molecular docking and synthesis of novel homopiperazine linked imidazo[1,2-a]pyrimidine derivatives as potent cytotoxic and antimicrobial agents. Bioorganic & Medicinal Chemistry Letters.

[ref20] (2003). Imidazopyrimidines, potent inhibitors of p38 MAP kinase. Bioorganic & Medicinal Chemistry Letters.

[ref21] (1994). Bicyclic imidazo derivatives, a new class of highly selective inhibitors for the human immunodeficiency virus type 1. Antiviral Research.

[ref22] (2015). Synthetic approaches and functionalizations of imidazo[1,2-a]pyrimidines: An overview of the decade. RSC Advances.

[ref23] (2020). Starch functionalized magnetite nanoparticles: A green, biocatalyst for one-pot multicomponent synthesis of imidazopyrimidine derivatives in aqueous medium under ultrasound irradiation. Journal of Molecular Structure.

[ref24] (1988). -a]pyrimidin-2-yl)phenylmethanones and related compounds as potential nonsedative anxiolytics. Journal of Medicinal Chemistry.

[ref25] (1989). Lack of anticonvulsant tolerance with RU 32698 and Ro 17-1812. European Journal of Pharmacology.

[ref26] (1991). - and 2-(thiazolyl)imidazo[1,2-a]pyrimidines as agonists and inverse agonists at benzodiazepine receptors. Journal of Medicinal Chemistry.

[ref27] (2020). Preparation of novel imidazo[1,2-a]pyrimidine derived schiff bases at conventional and microwave heating conditions. Journal of Balıkesir University Institute of Science and Technology.

[ref28] (2020). One pot, multicomponent protocol for the synthesis of novel imidazo[1,2‑a]pyrimidine‑based pyran analogs: A potential biological scaffold. Monatshefte für Chemi-Chemical Monthly.

[ref29] (2018). Co(II) anchored glutaraldehyde crosslinked magnetic chitosan nanoparticles (MCS) for synthesis of 2,4,5‐trisubstituted and 1,. Applied Organometallic Chemistry.

[ref30] (2004). Efficient synthesis of imidazoles from aldehydes and 1,2-diketones using microwave irradiation. Organic Letters.

[ref31] (2018). An eco-compatible pathway to the synthesis of mono and bis-multisubstituted imidazoles over novel reusable ionic liquids: An efficient and green sonochemical process. RSC Advances.

[ref32] (2008). Highly efficient, four component, one-pot synthesis of tetrasubstituted imidazoles using a catalytic amount of FeCl_3_.6H_2_O. Monatshefte für Chemie.

[ref33] (2010). One-pot synthesis of tri- and tetra-substituted imidazoles using sodium dihydrogen phosphate under solvent-free conditions. Chinese Chemical Letters.

[ref34] (2010). Four-component, one-pot synthesis of tetra-substituted imidazoles using a catalytic amount of MCM-41 or p-TsOH. Synthetic Communications.

[ref35] (2013). Simple and efficient method for the synthesis of highly substituted imidazoles catalyzed by benzotriazole. Journal of Heterocyclic Chemistry.

[ref36] (2012). A facile strategy for the synthesis of highly substituted imidazole using tetrabutyl mmoniumbromide as catalyst. Research on Chemical Intermediates.

[ref37] (2018). Cu(II) immobilized on guanidinated epibromohydrinfunctionalized γ‐Fe_2_O_3_@TiO_2_(γ‐Fe_2_O_3_@TiO_2_‐EG‐Cu(II)): A highly efficient magnetically separable heterogeneous nanocatalyst for one‐pot synthesis of highly substituted imidazoles. Applied Organometallic Chemistry.

[ref38] (2016). A new more atom-efficient multi-component approach to tetrasubstituted imidazoles: one-pot condensation of nitriles, amines and benzoin. RSC Advances.

[ref39] (2009). Controlled microwave heating in modern organic synthesis: highlights from the 2004–2008 literature. Molecular Diversity.

[ref40] (2011). A critical assessment of the greenness and energy efficiency of microwave-assisted organic synthesis. Green Chemistry.

[ref41] (u26b9). The use of microwave ovens for rapid organic synthesis. u26b5.

[ref42] (1986). Application of commercial microwave ovens to organic synthesis. u26b2.

[ref43] (2001). Microwave assisted organic synthesis-a review. Tetrahedron.

[ref44] (2018). Green synthesis of new tetra schiff bases and bis-azo bis-schiff bases derived from 2,6-diaminopyridine as promising photosensitizers. International Journal of Organic Chemistry.

[ref45] (2019). Microwave assisted synthesis of novel schiff bases of pyrazolyl carbaldehyde and triazole in. PEG-400. Polycyclic Aromatic Compounds.

[ref46] (2005). One‐step synthesis of 2‐aryl‐4,5‐diphenylimidazoles under microwave irradiation. Synthetic Communications.

[ref47] (2012). One-pot synthesis of polysubstituted imidazoles from arylaldehydes in water catalyzed by NHC using microwave irradiation. Journal of the Chilean Chemical Society.

[ref48] (2020). Review on multi-component green synthesis of N-containing heterocycles using mixed oxides as heterogeneous catalysts. Arabian Journal of Chemistry.

[ref49] (2018). Toward the synthesis of Sceptrin and Benzosceptrin: Solvent effect in stereo-. regioselective [2+.

[ref50] (2009). Microwave-assisted, solvent-free, parallel syntheses and elucidation of reaction mechanism for the formation of some novel tetraaryl imidazoles of biological interest. Journal of Heterocyclic Chemistry.

