# Supplementary material for: Microwave assisted, sequential two-step, one-pot synthesis of novel imidazo[1,2-a]pyrimidine containing tri/tetrasubstituted imidazole derivatives
Source: Turk J Chem. 2021 Feb 17;45(1):219–30. doi: 10.3906/kim-2009-40 (PMC7925308; doi:10.3906/kim-2009-40)
Supplement: Supplementary file 1 — Supplementary Materials [file turkjchem-45-219-sup001.pdf]

Supporting Information

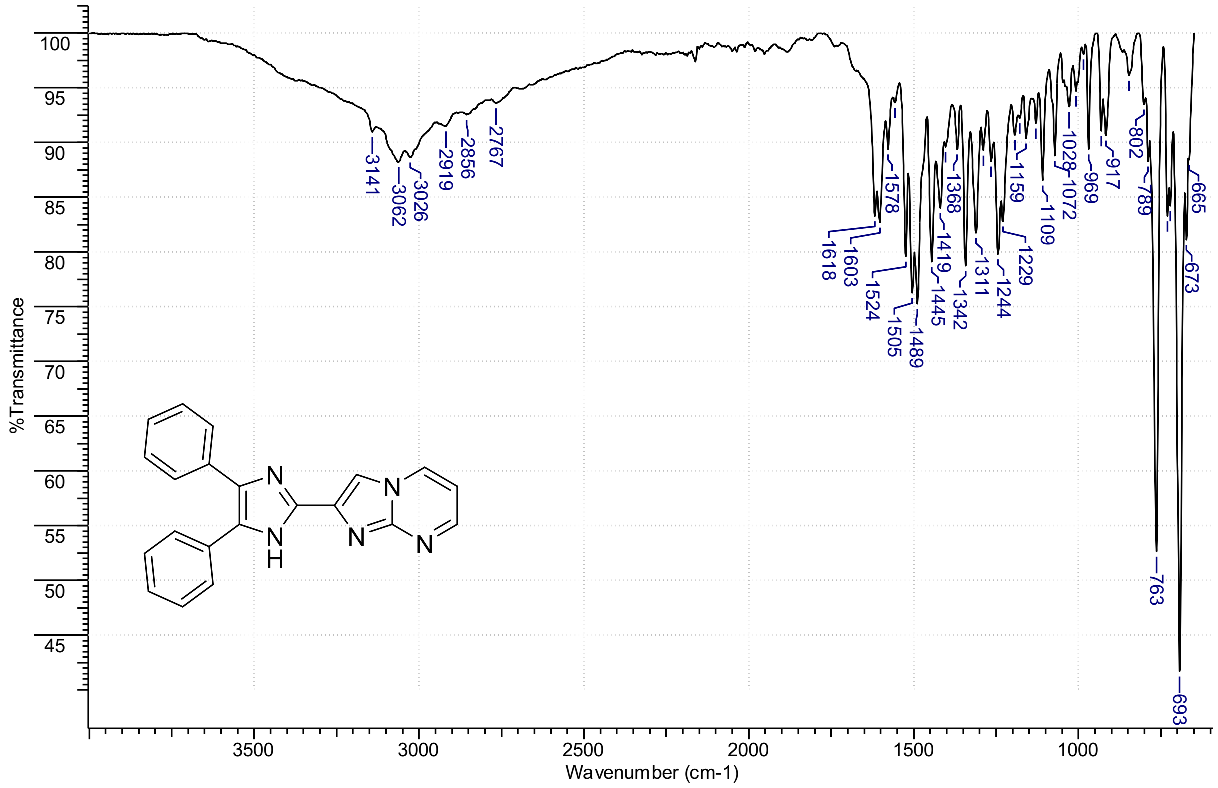

Figure S1. FT-IR spectra of compound 1.

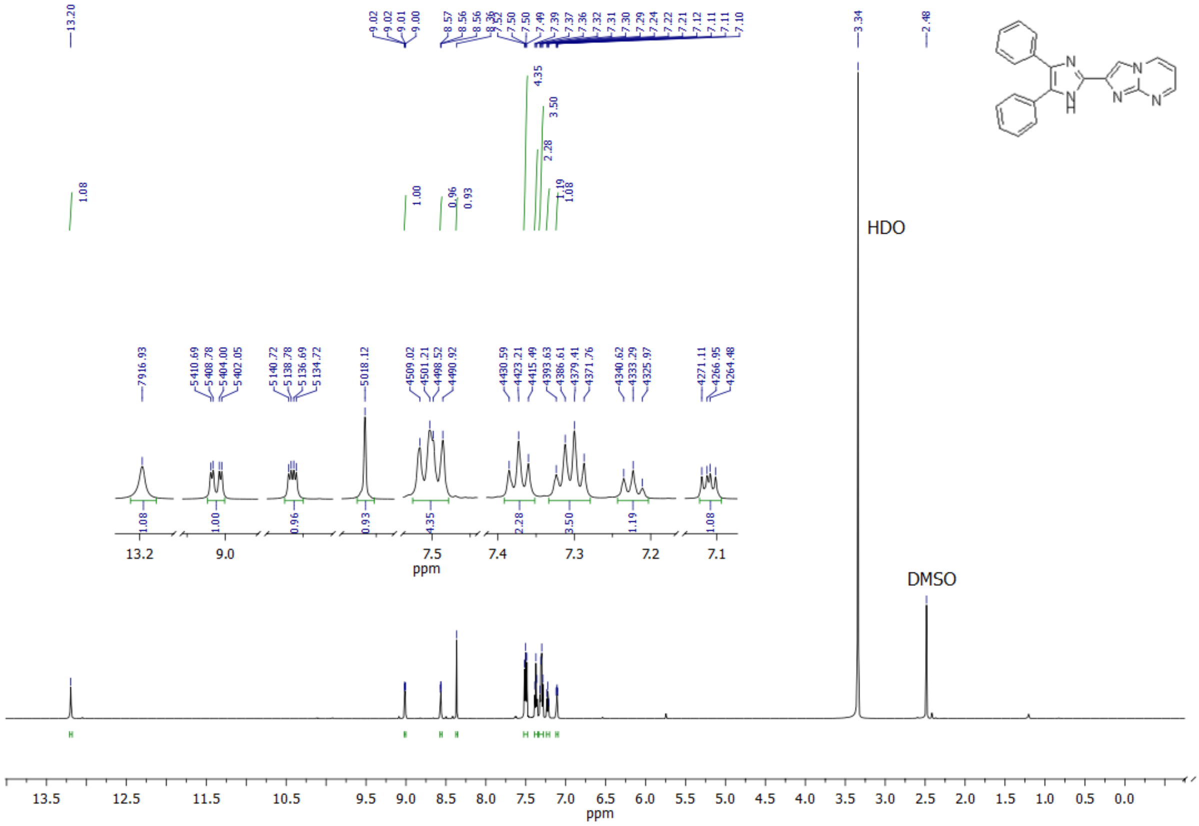

Figure S2. ^{1}H NMR spectra of compound 1.

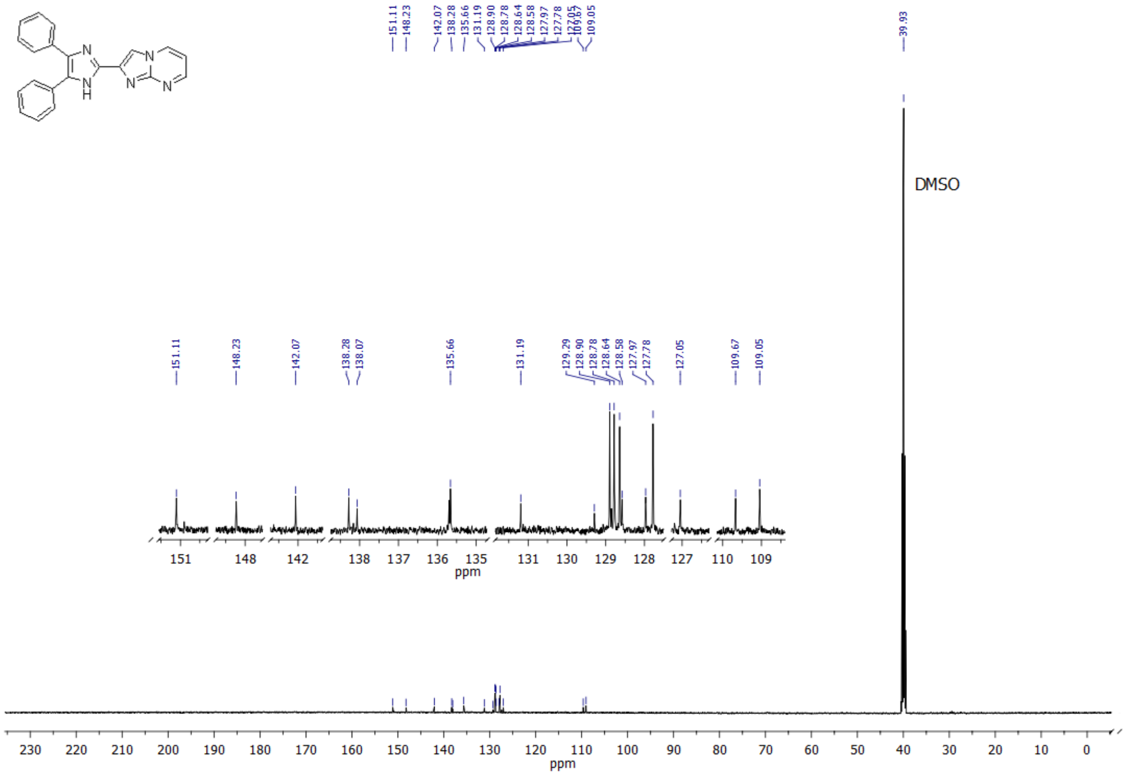

Figure S3. ^{13}C NMR spectra of compound 1.

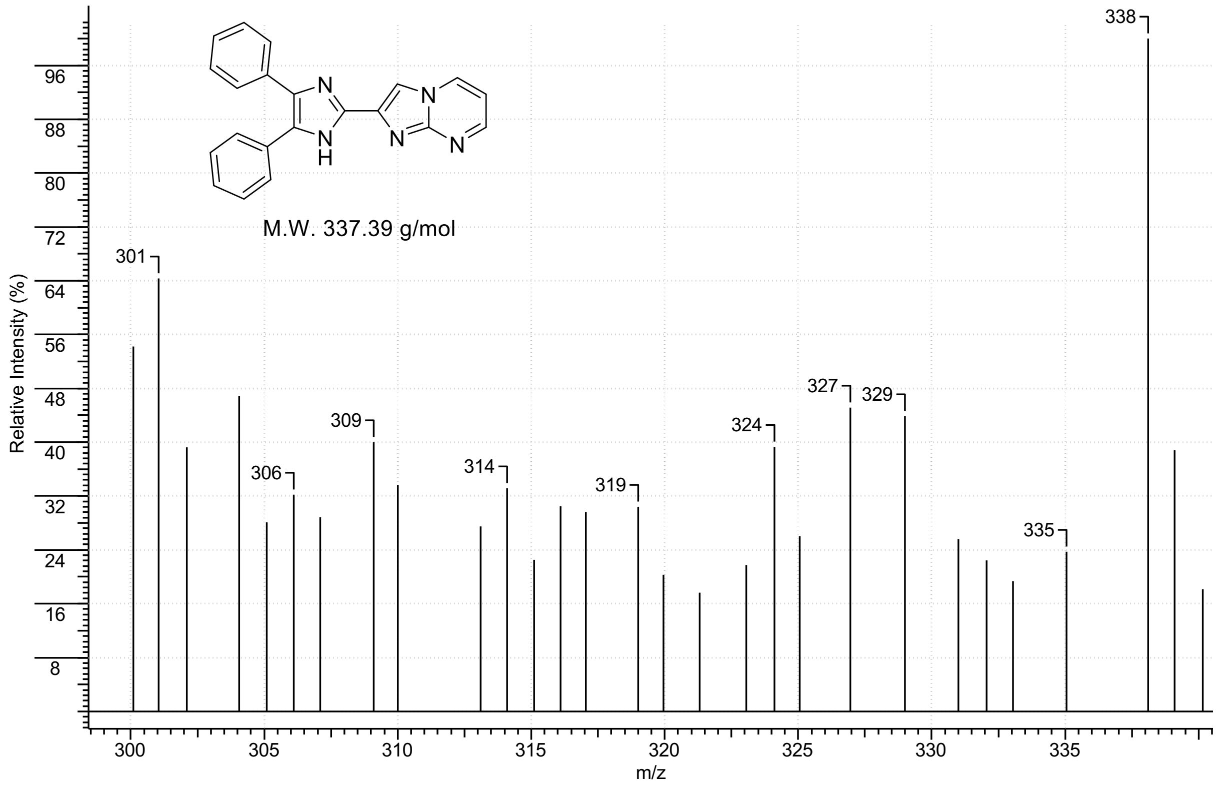

Figure S4. MS spectra of compound 1.

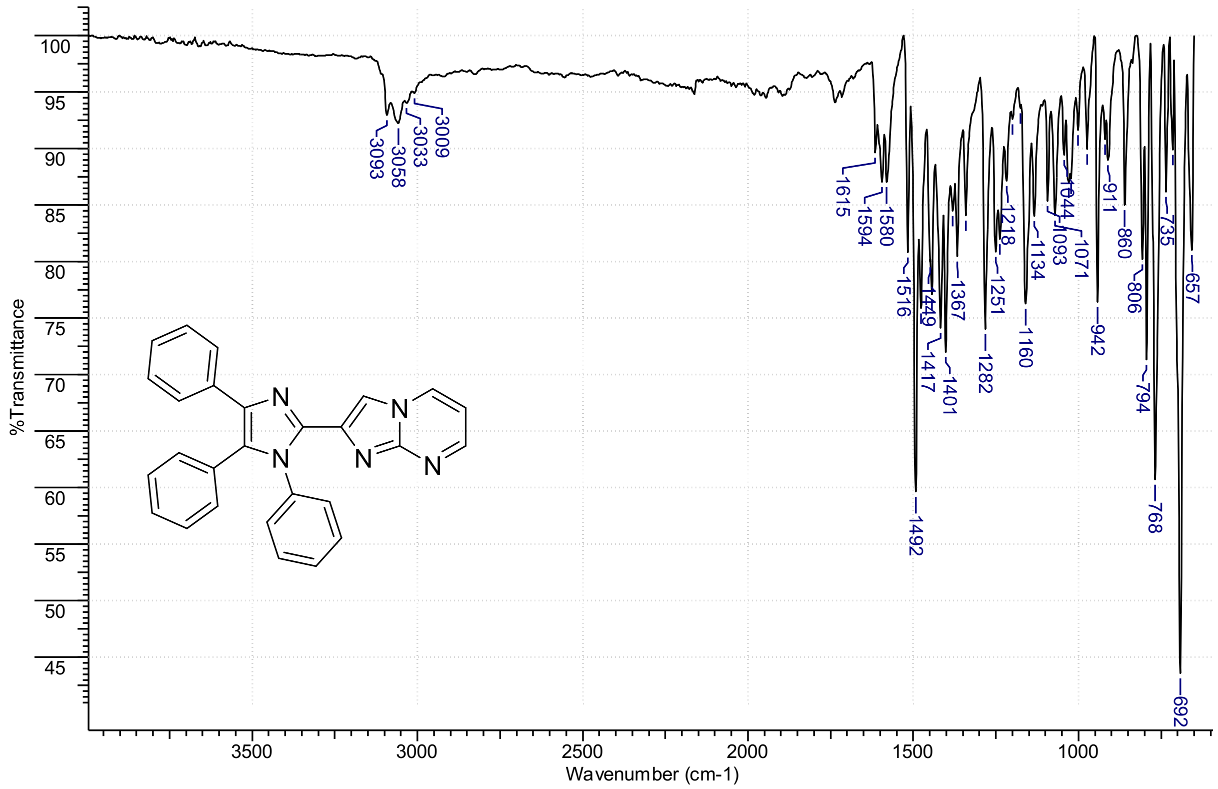

Figure S5. FT-IR spectra of compound 2.

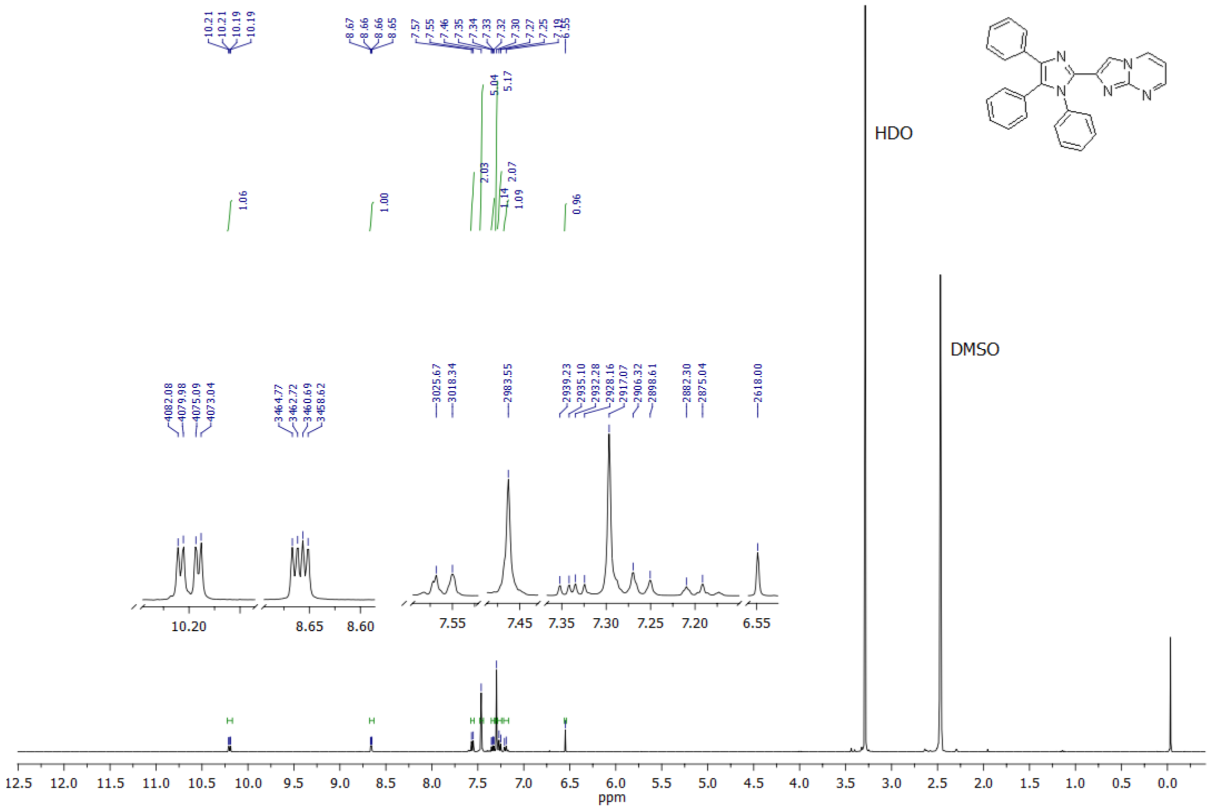

Figure S6. ^{1}H NMR spectra of compound 2.

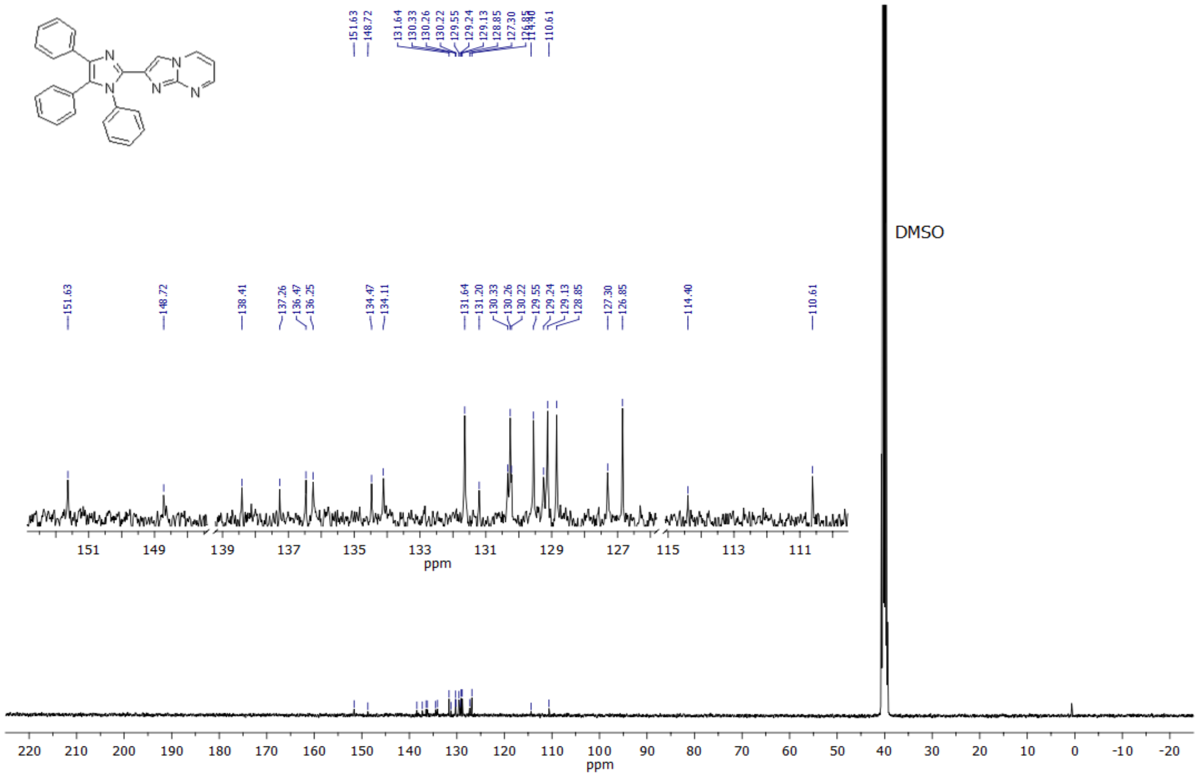

Figure S7. ^{13}C NMR spectra of compound 2.

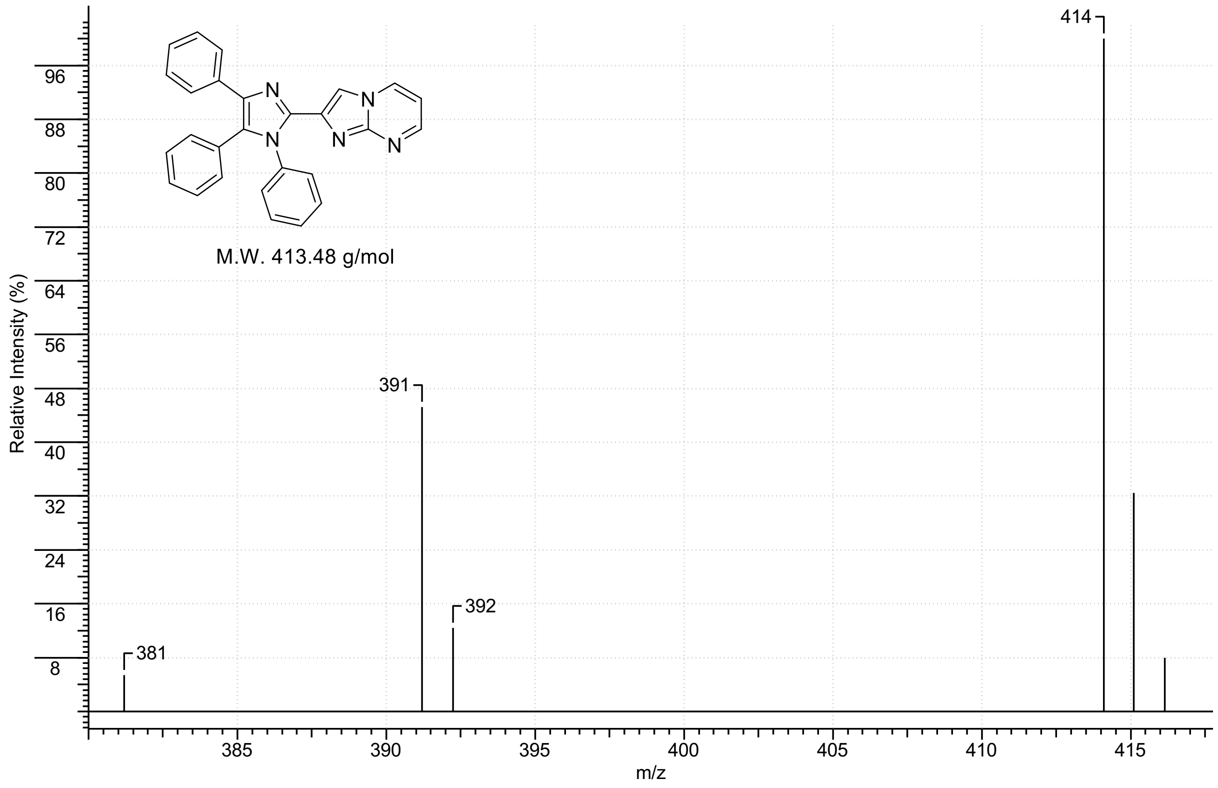

Figure S8. MS spectra of compound 2.

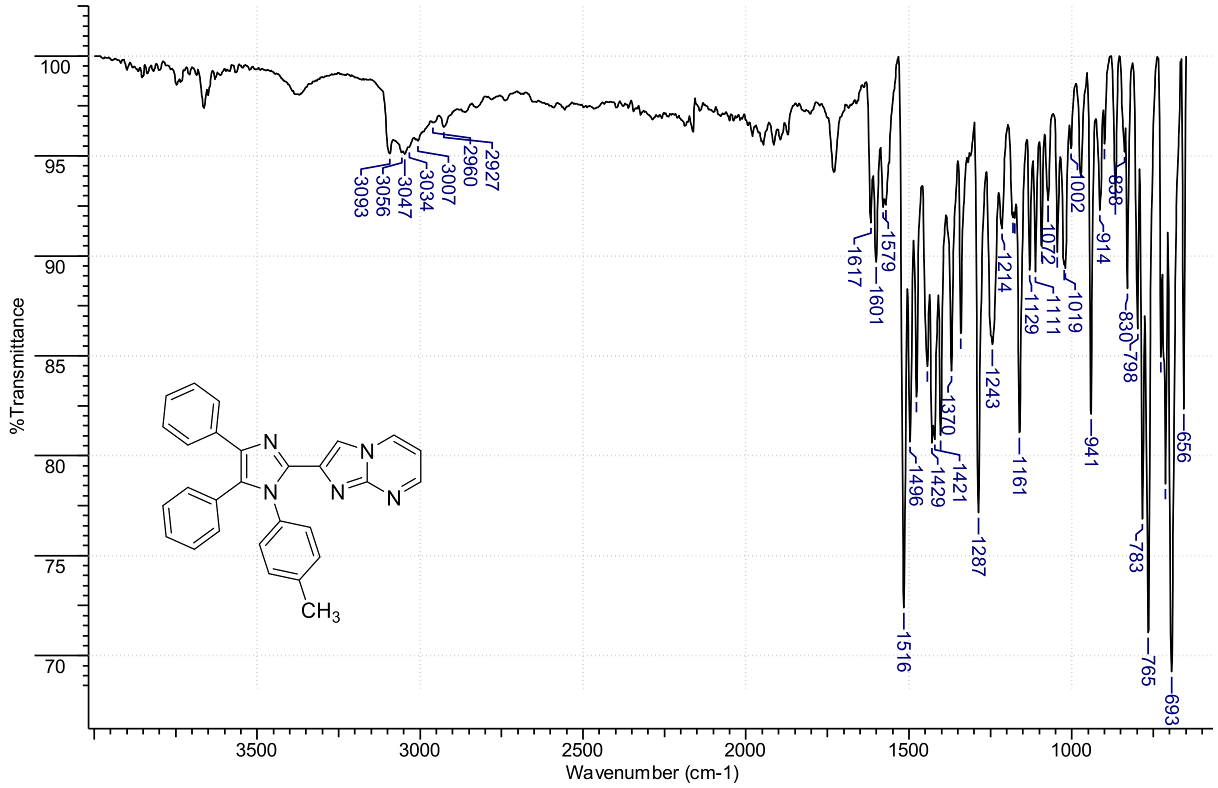

Figure S9. FT-IR spectra of compound 3.

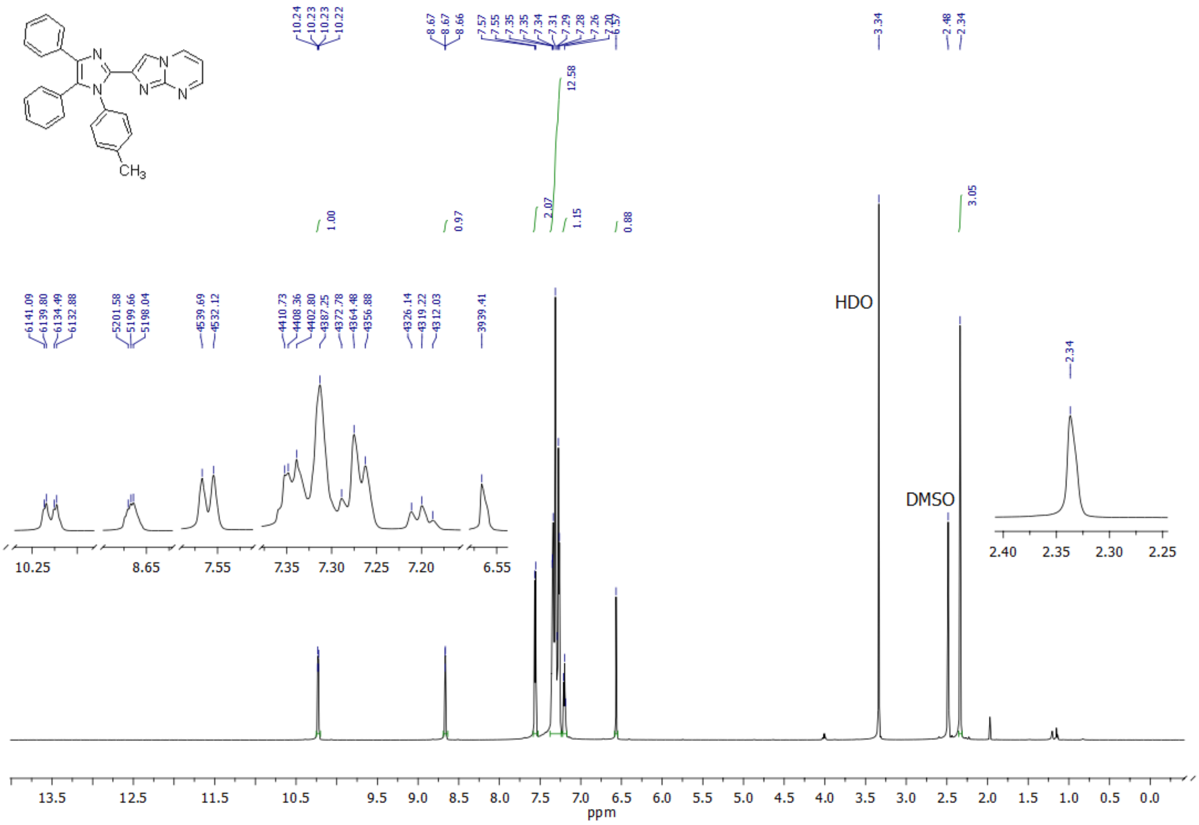

Figure S10. ^{1}H NMR spectra of compound 3.

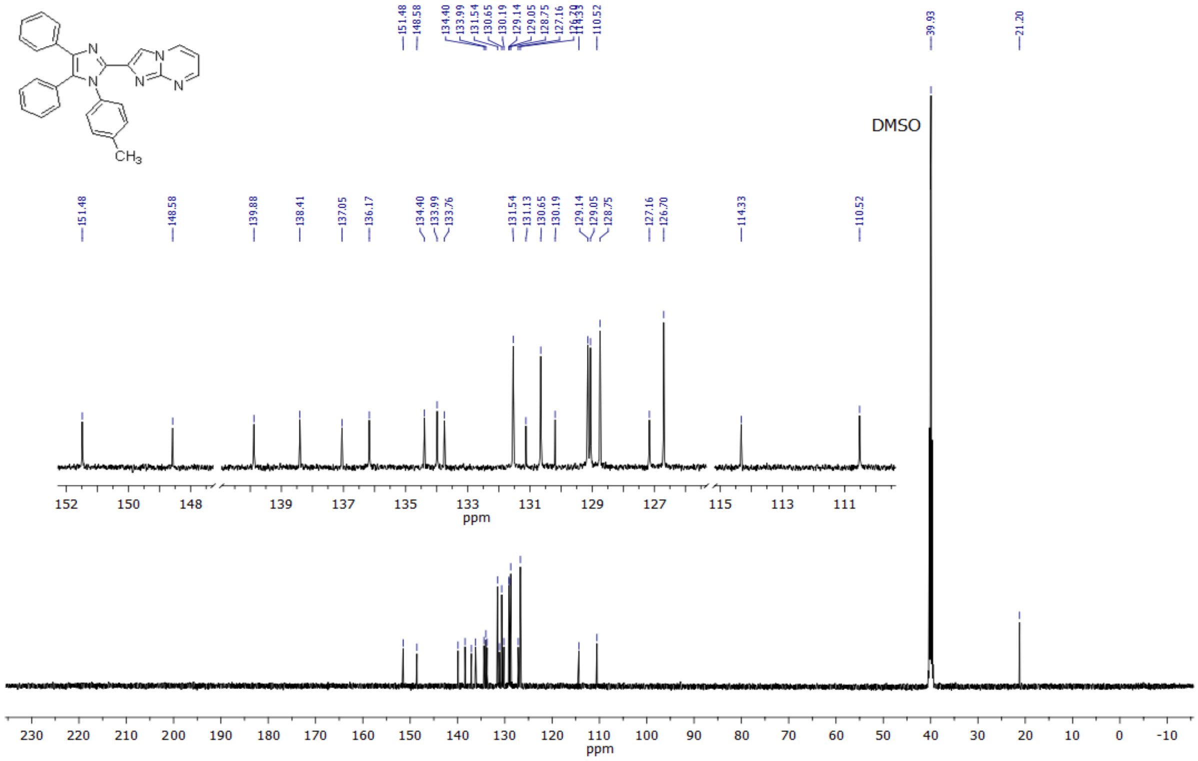

Figure S11. ^{13}C NMR spectra of compound 3.

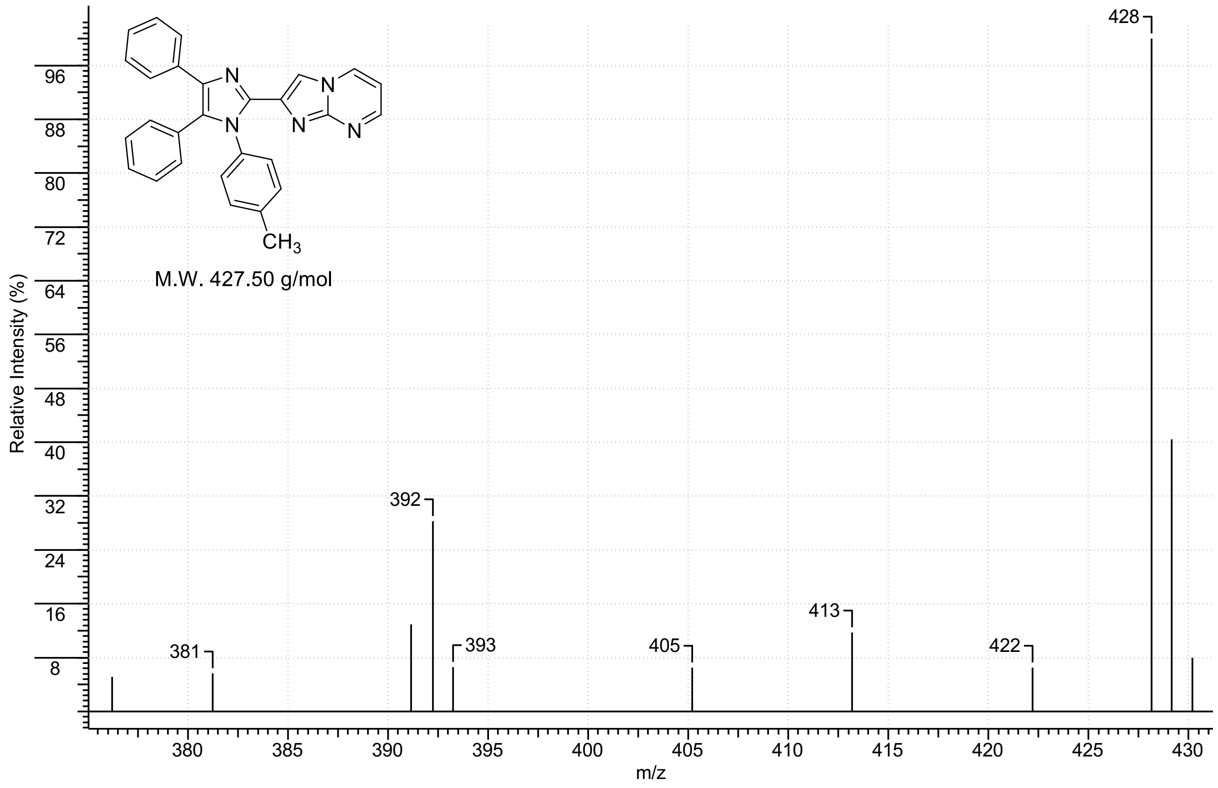

Figure S12. MS spectra of compound 3**.**

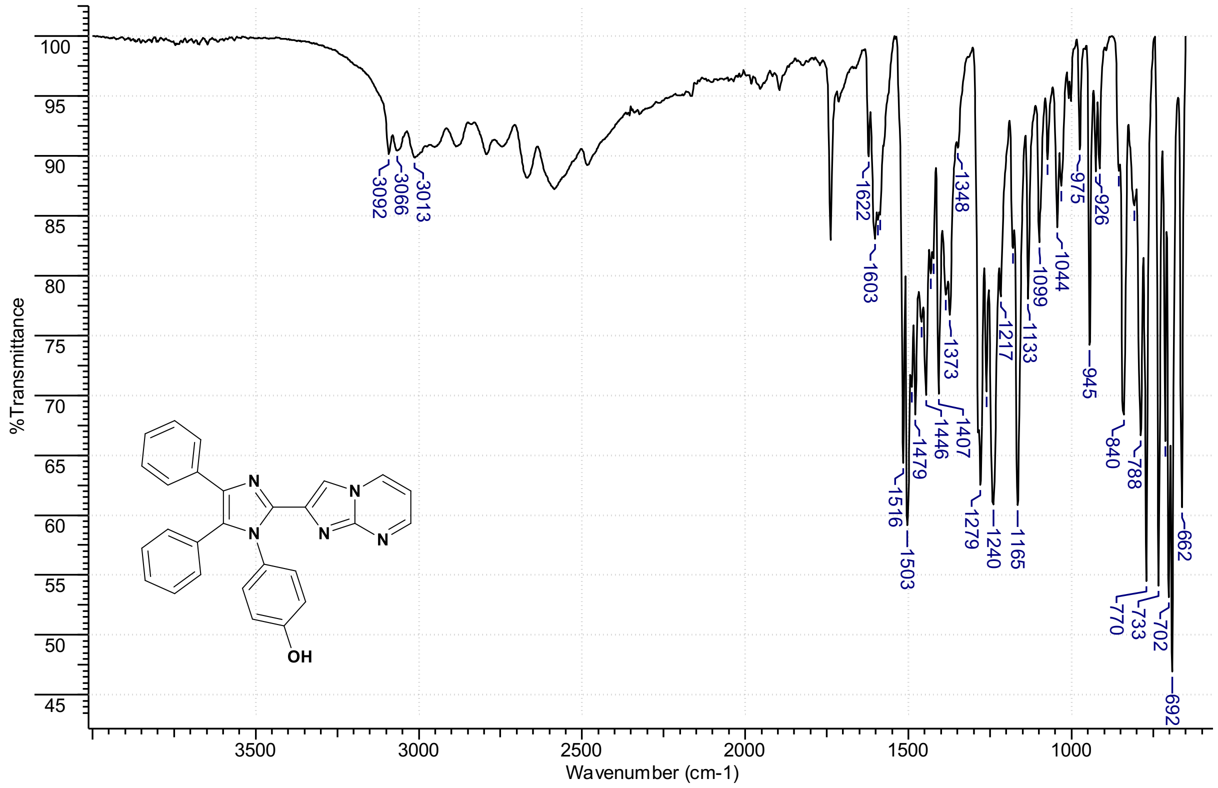

Figure S13. FT-IR spectra of compound 4.

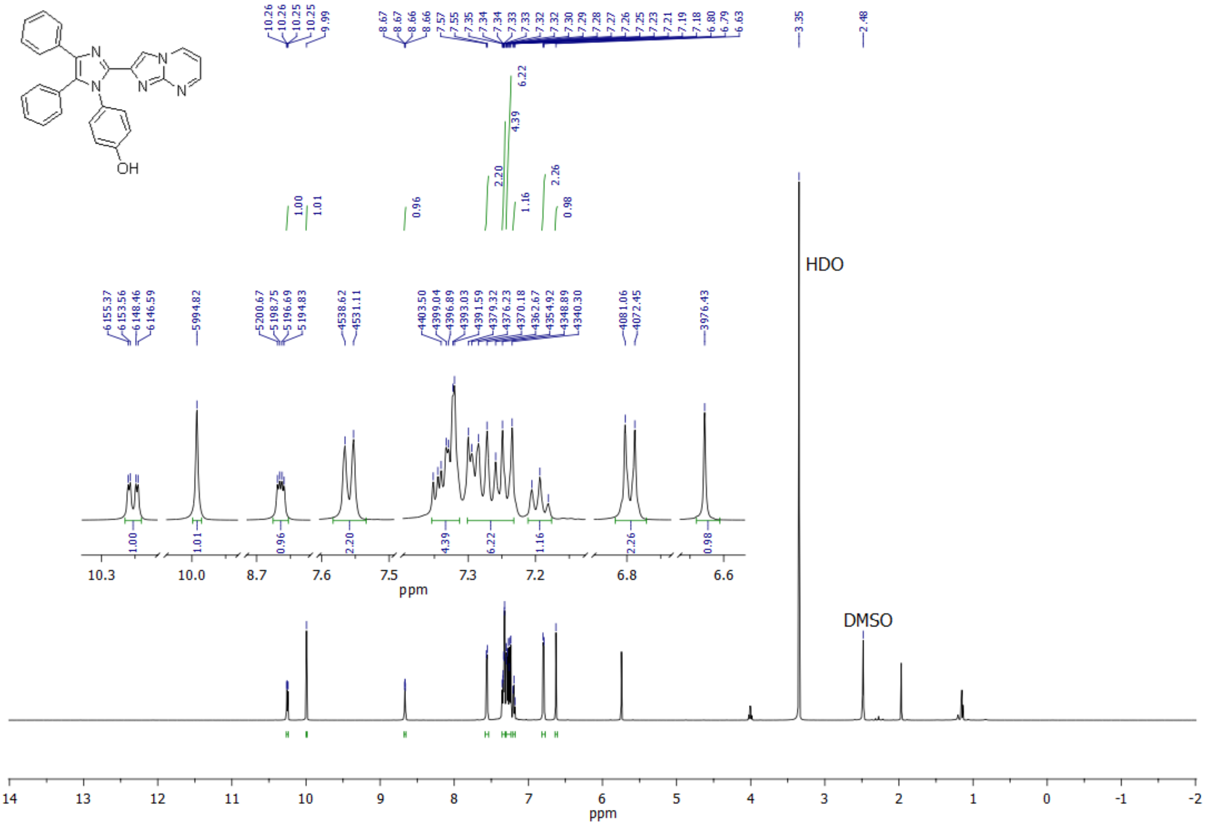

Figure S14. ^{1}H NMR spectra of compound 4.

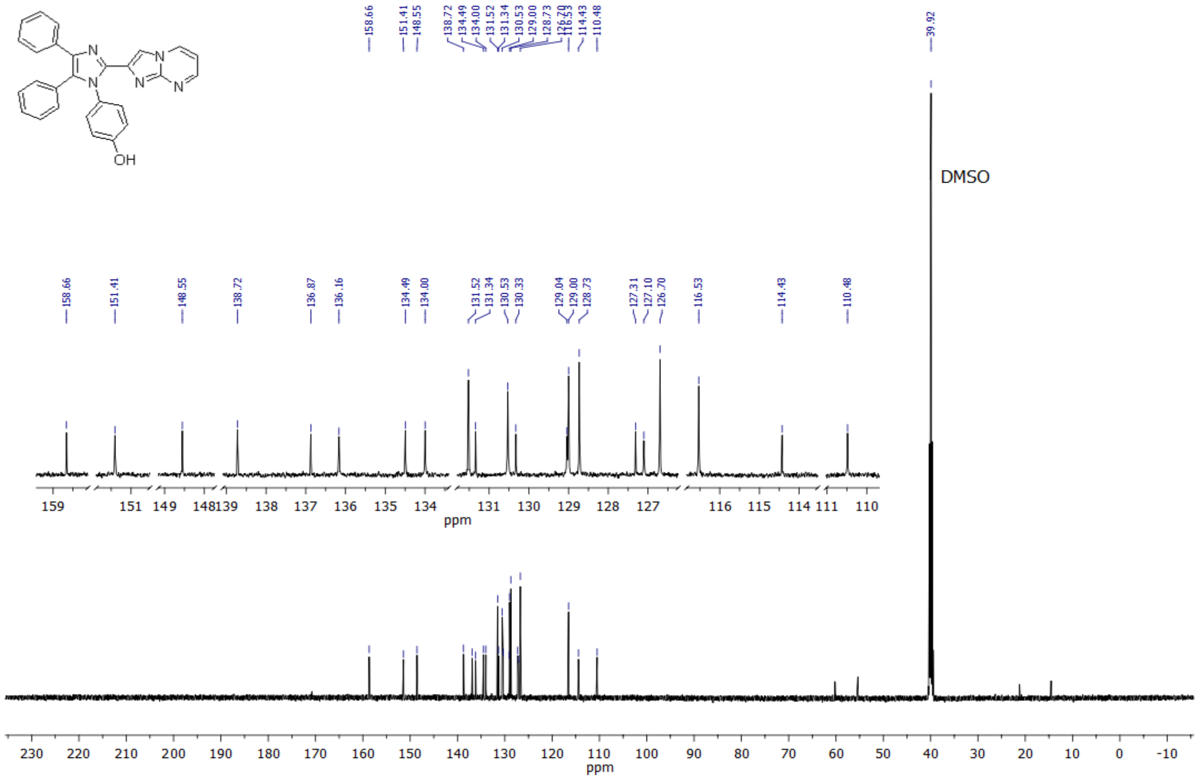

Figure S15. ^{13}C NMR spectra of compound 4.

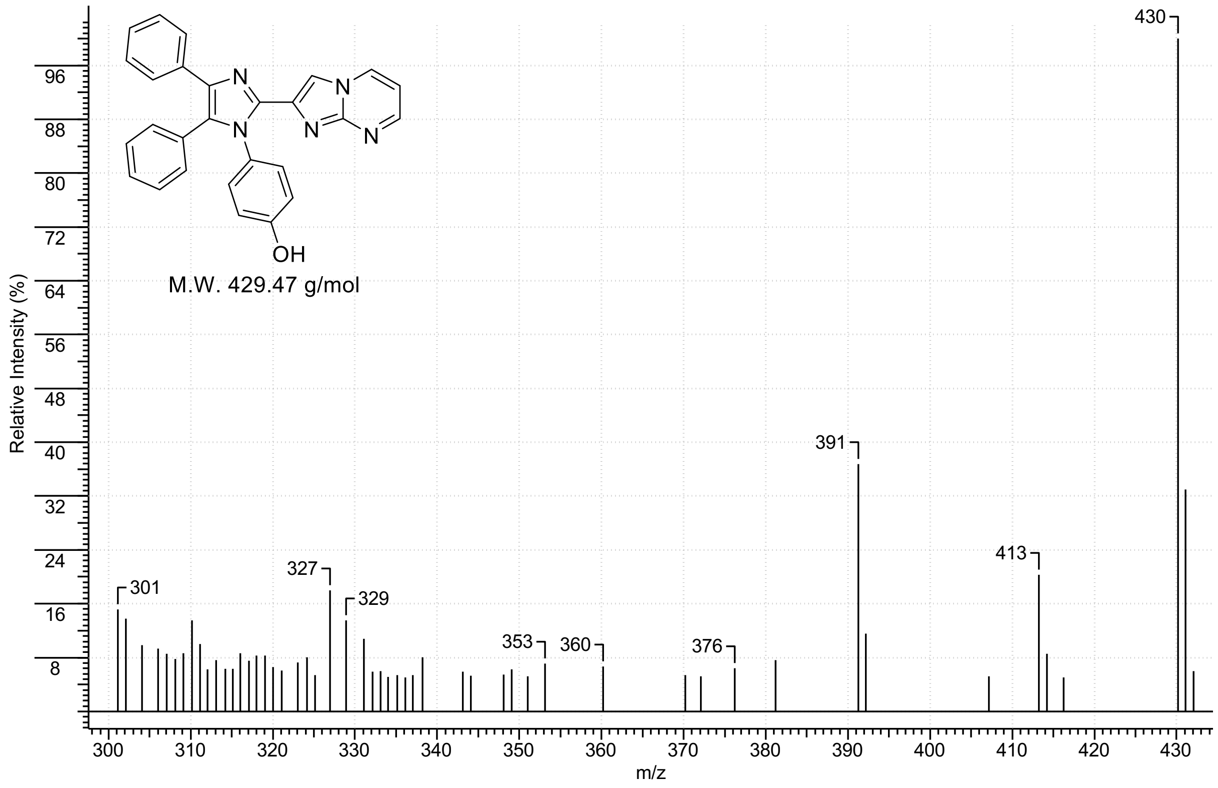

Figure S16. MS spectra of compound 4.

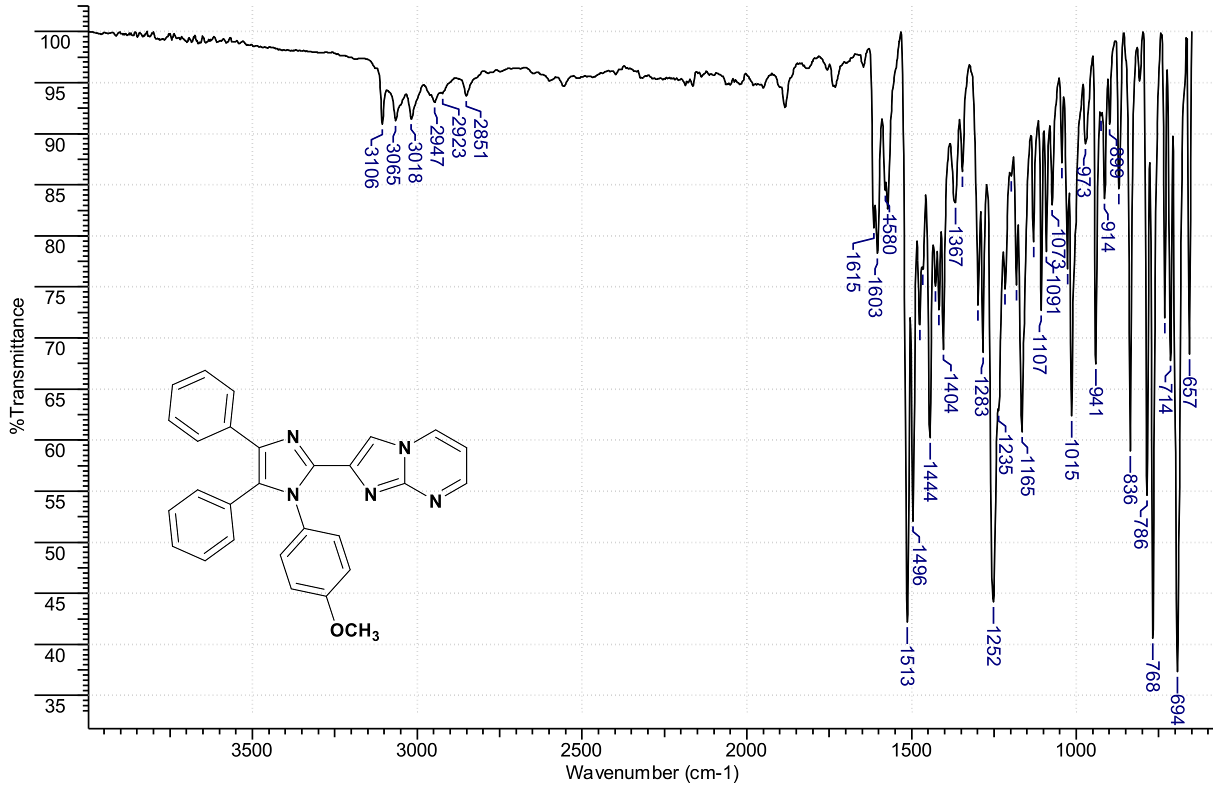

Figure S17. FT-IR spectra of compound 5.

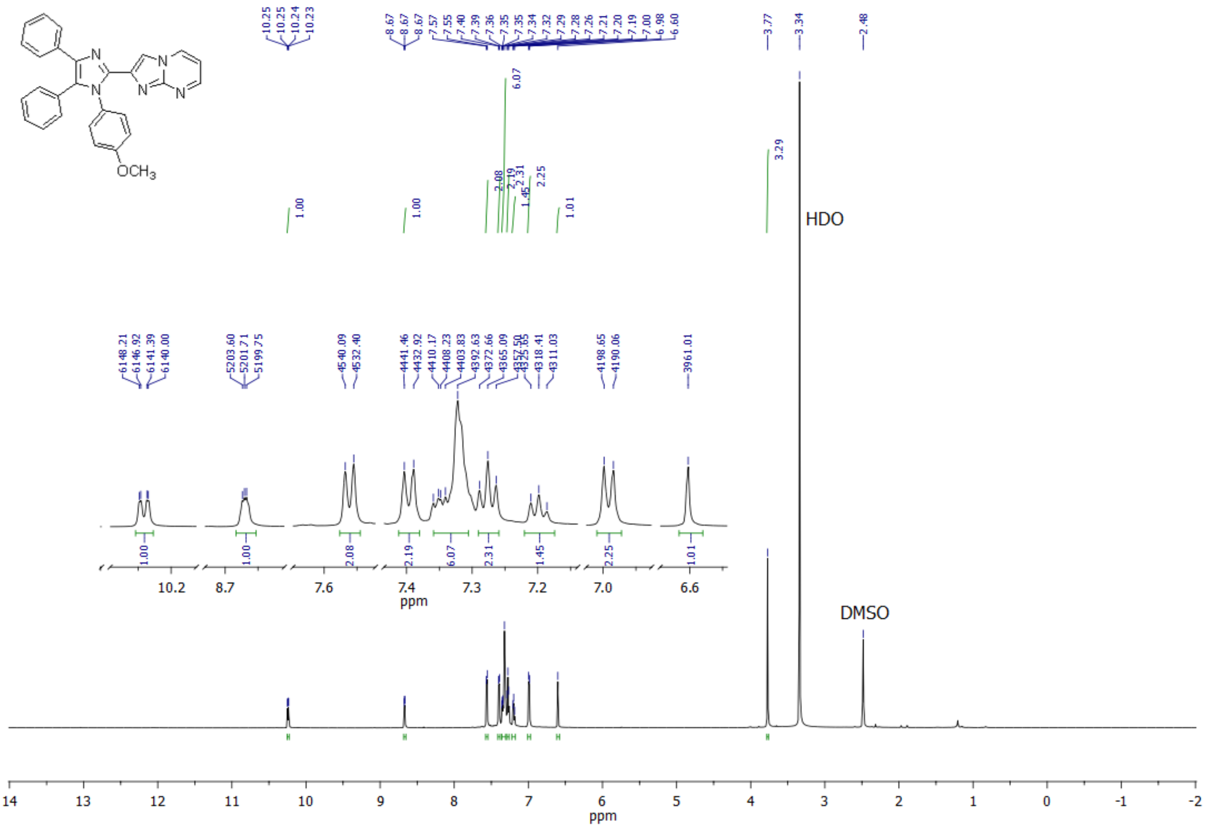

Figure S18. ^{1}H NMR spectra of compound 5.

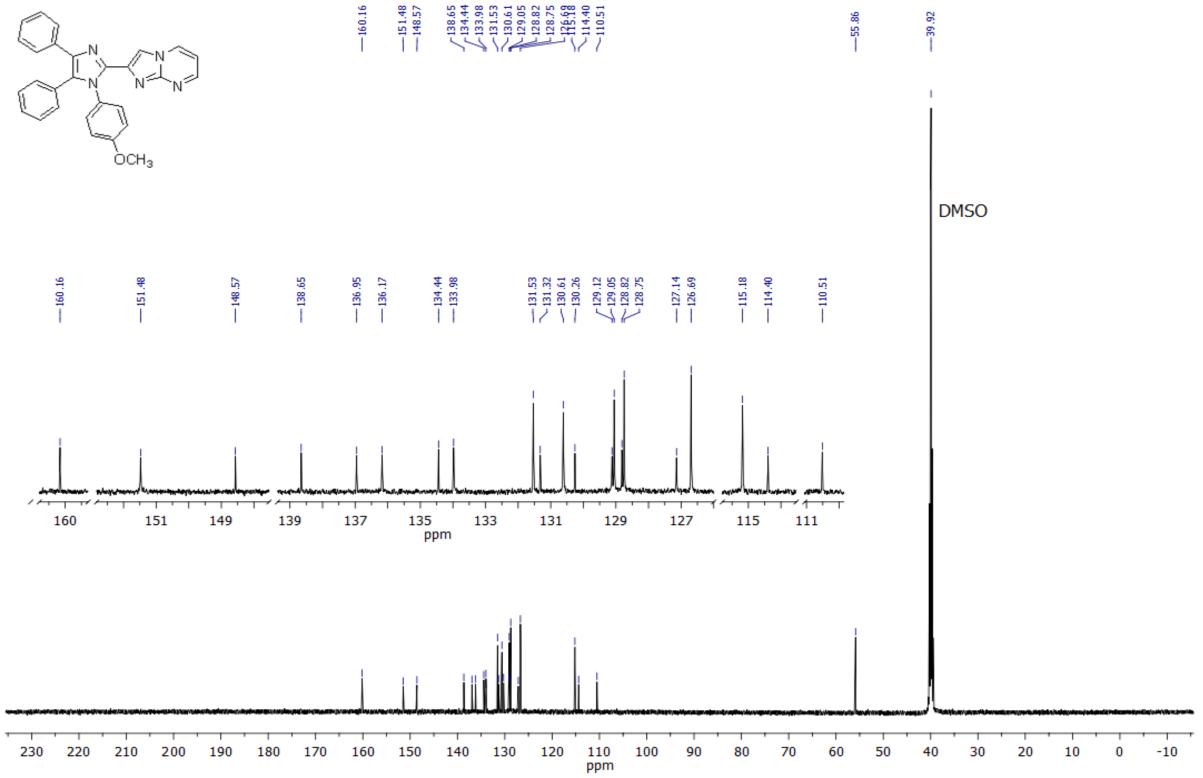

Figure S19. ^{13}C NMR spectra of compound 5.

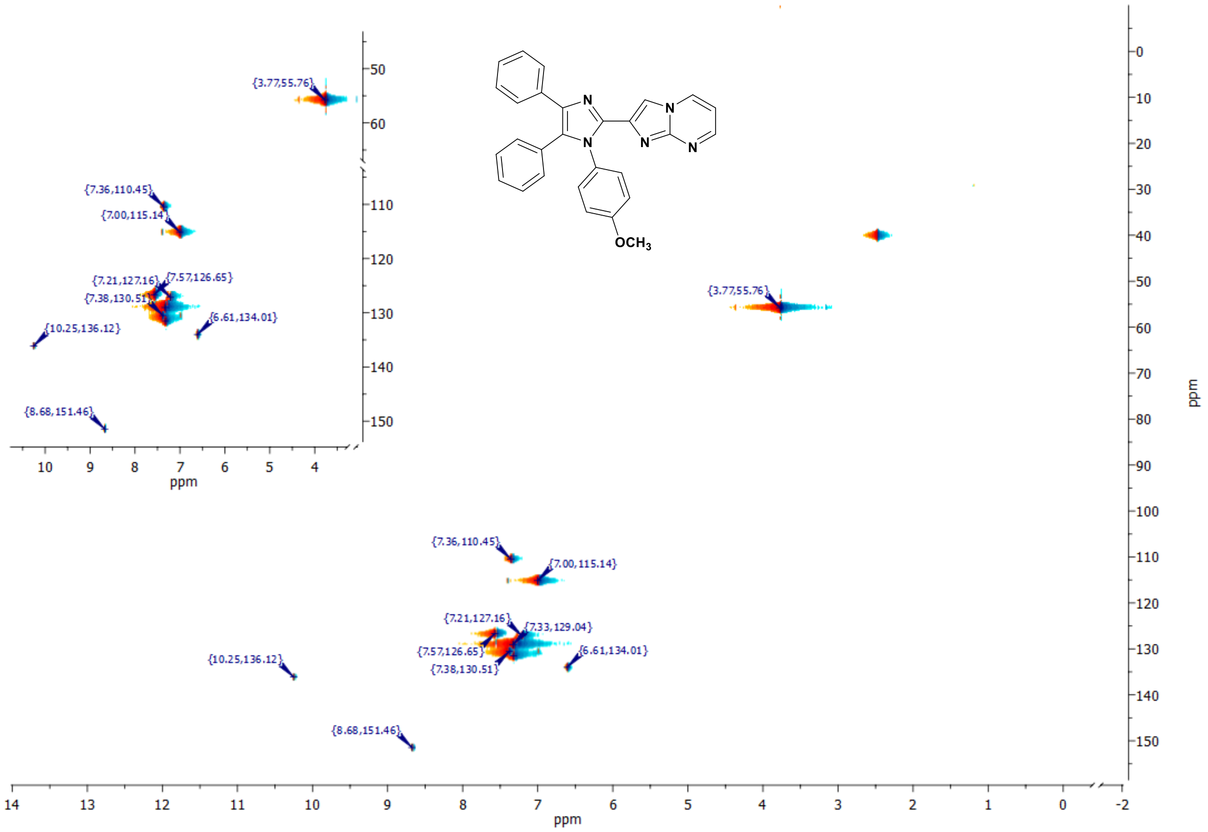

Figure S20. HSQC spectra of compound 5.

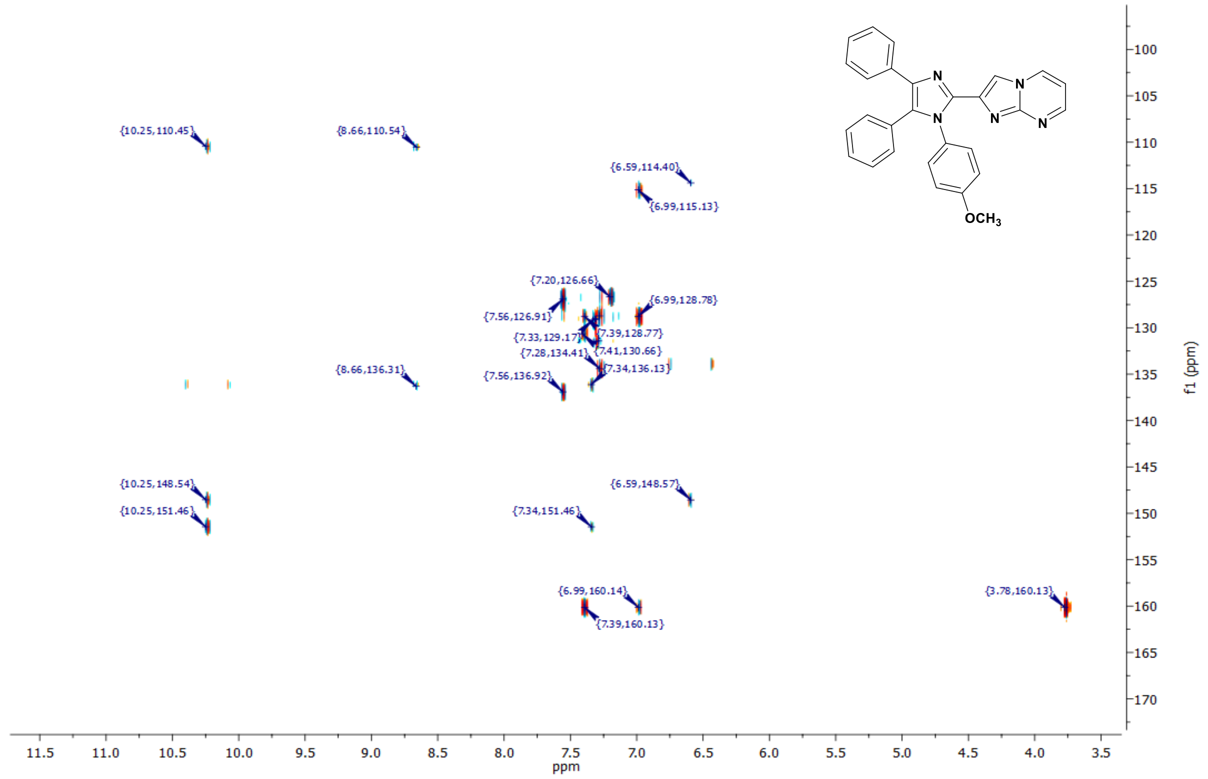

Figure S21. HMBC spectra of compound 5.

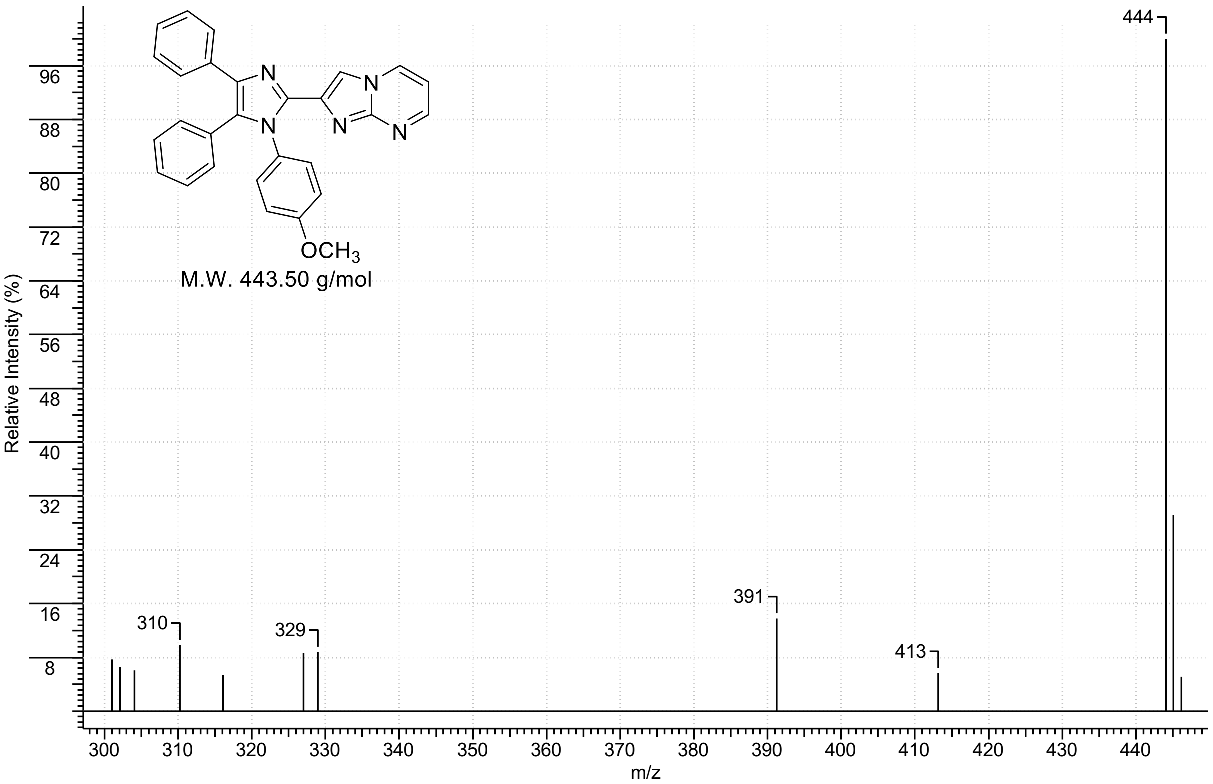

Figure S22. MS spectra of compound 5.

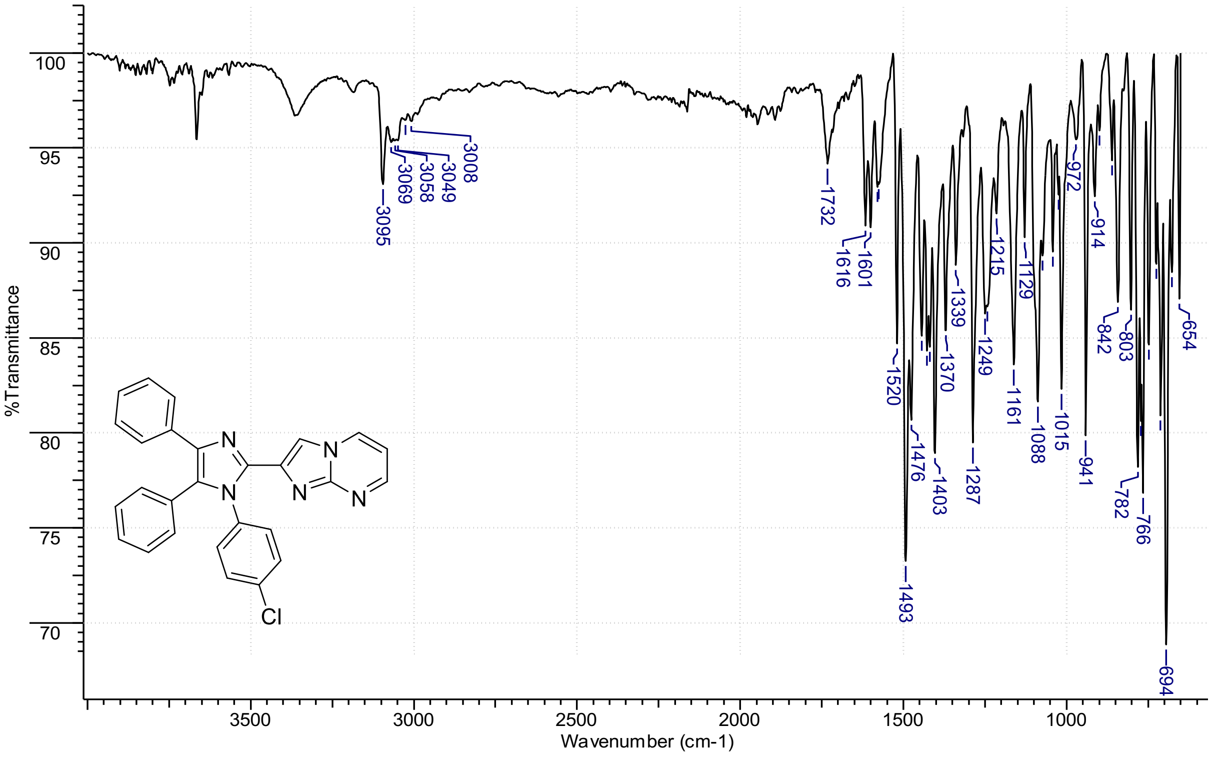

Figure S23. FT-IR spectra of compound 6.

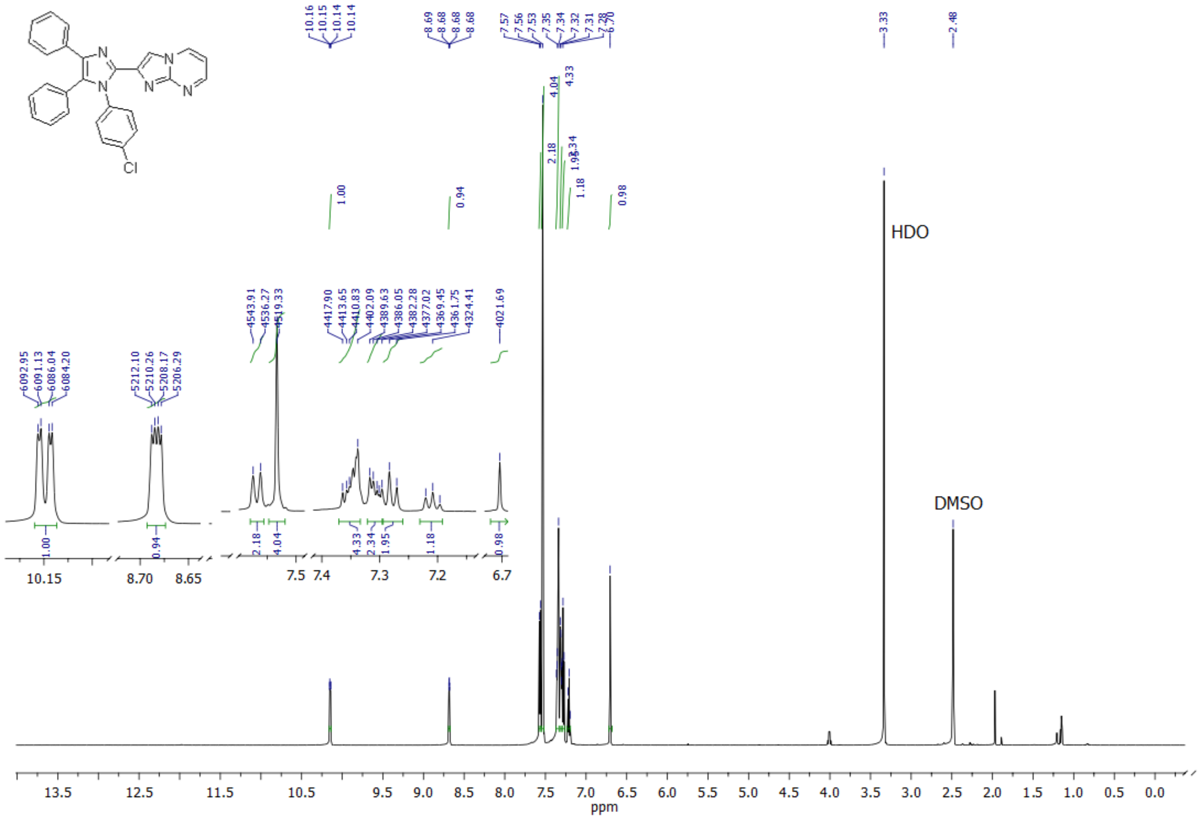

Figure S24. ^{1}H NMR spectra of compound 6.

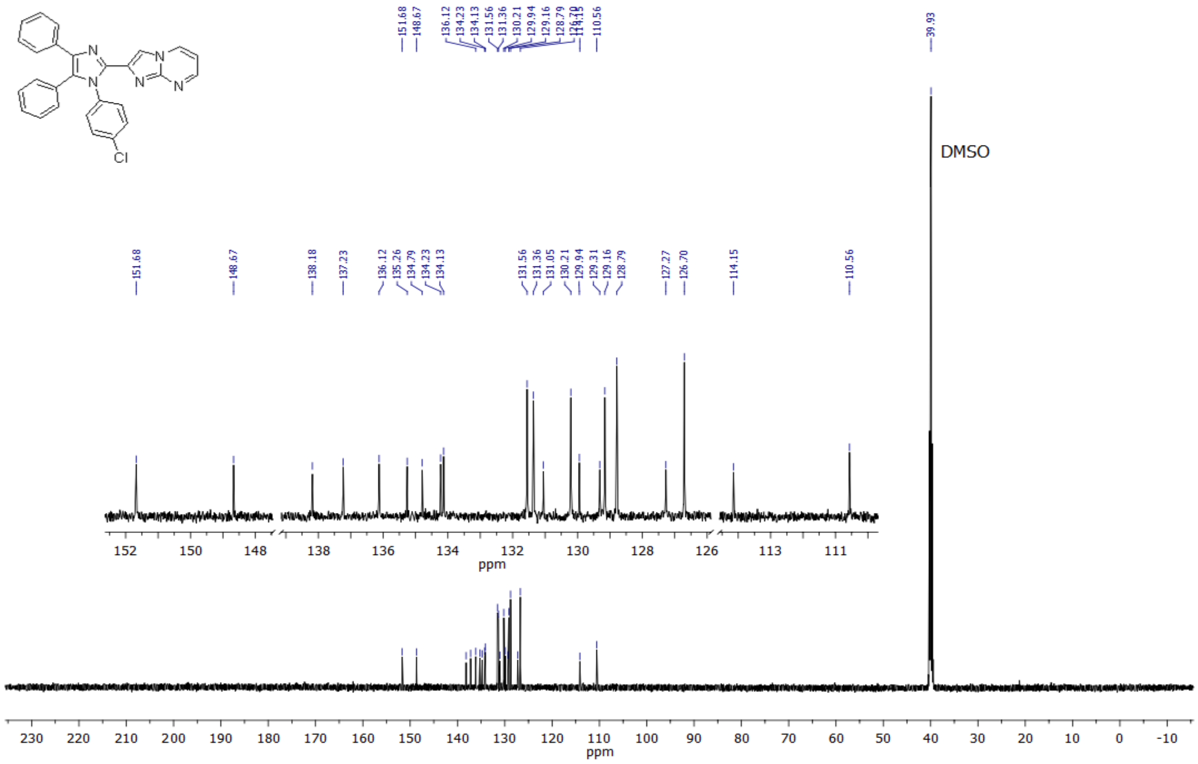

Figure S25. ^{13}C NMR spectra of compound 6.

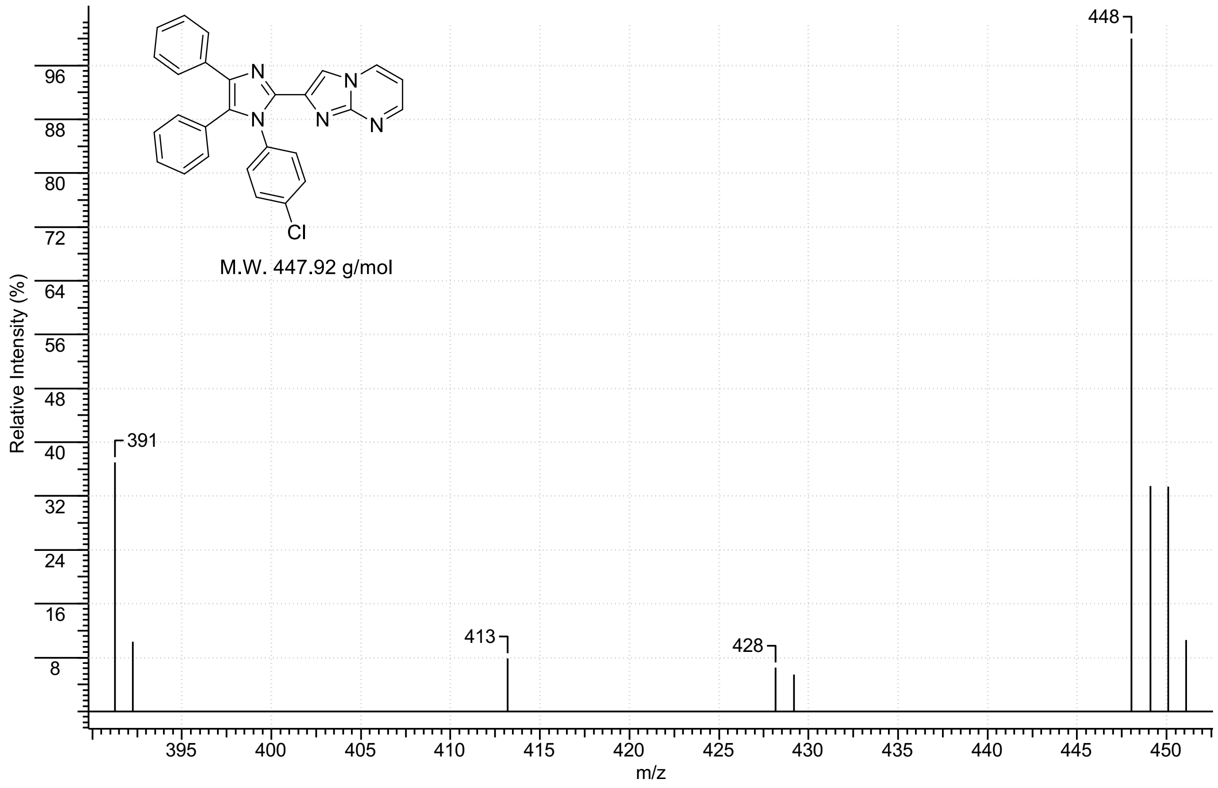

Figure S26. MS spectra of compound 6.

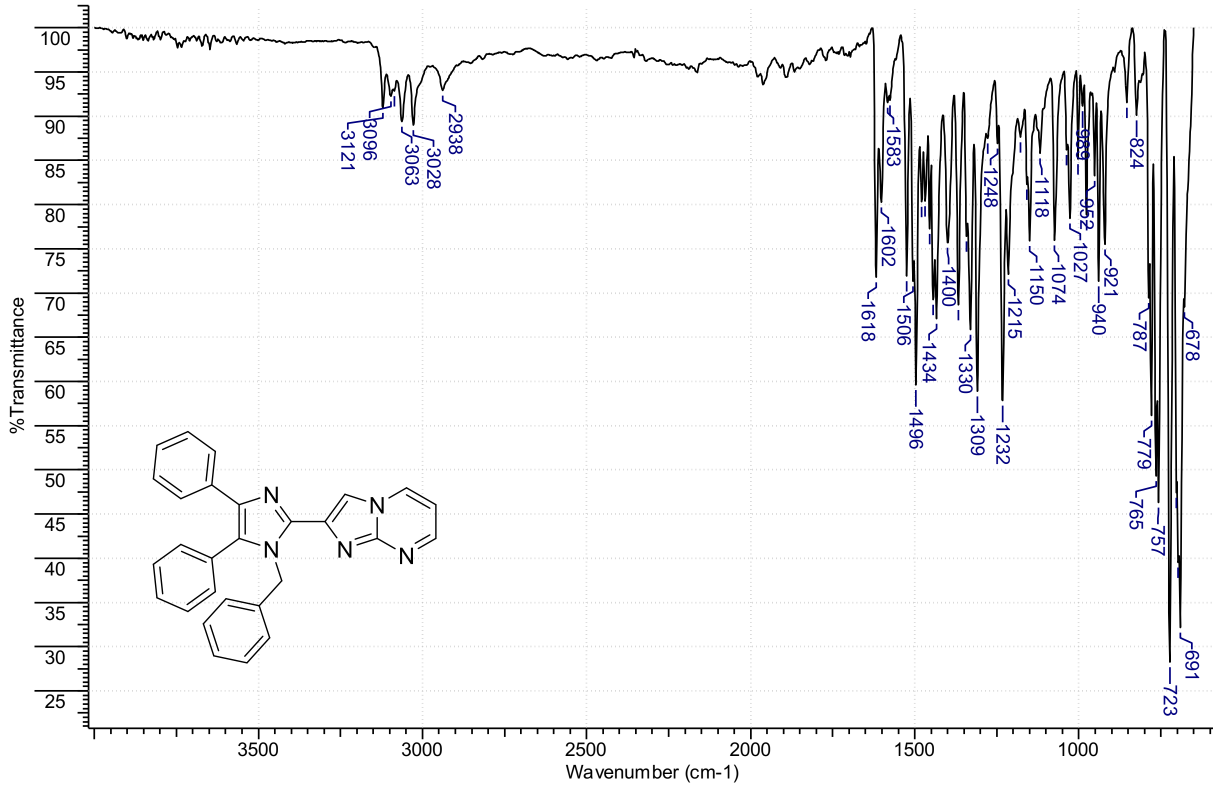

Figure S27. FT-IR spectra of compound **7**.

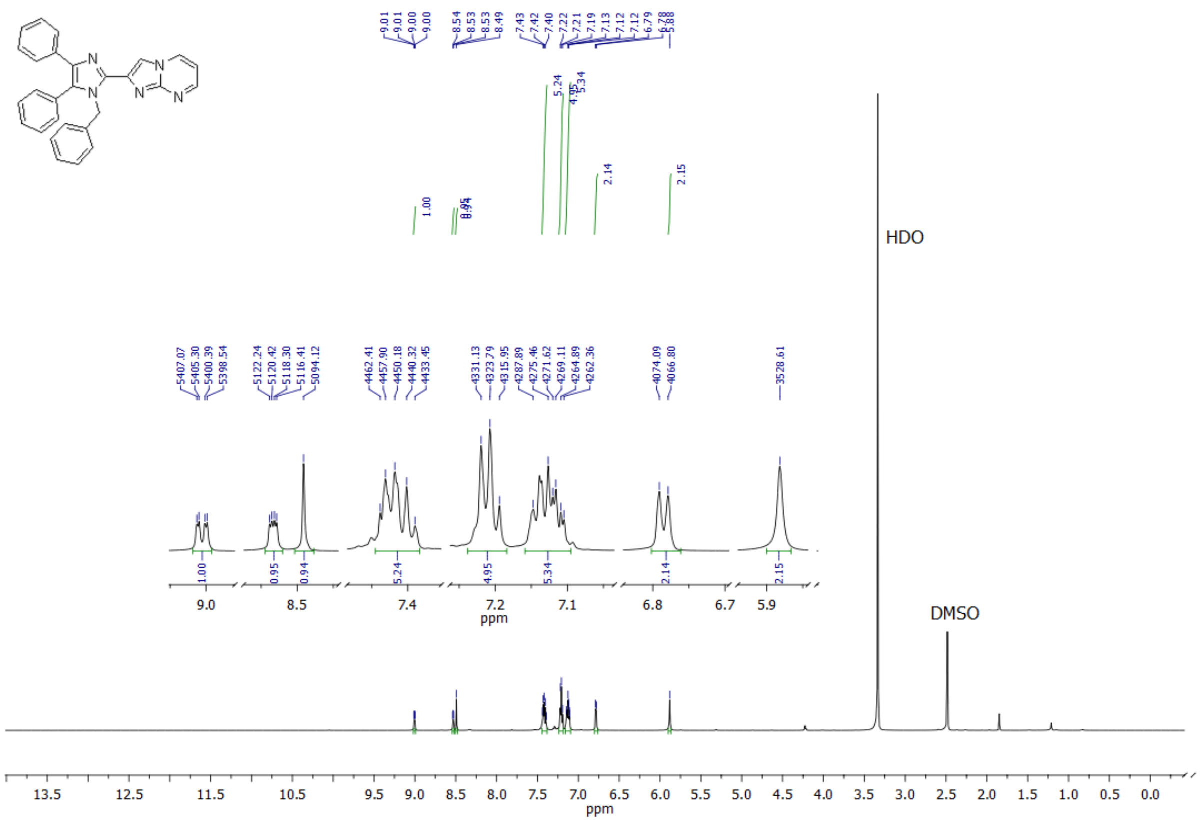

Figure S28. ^{1}H NMR spectra of compound **7**.

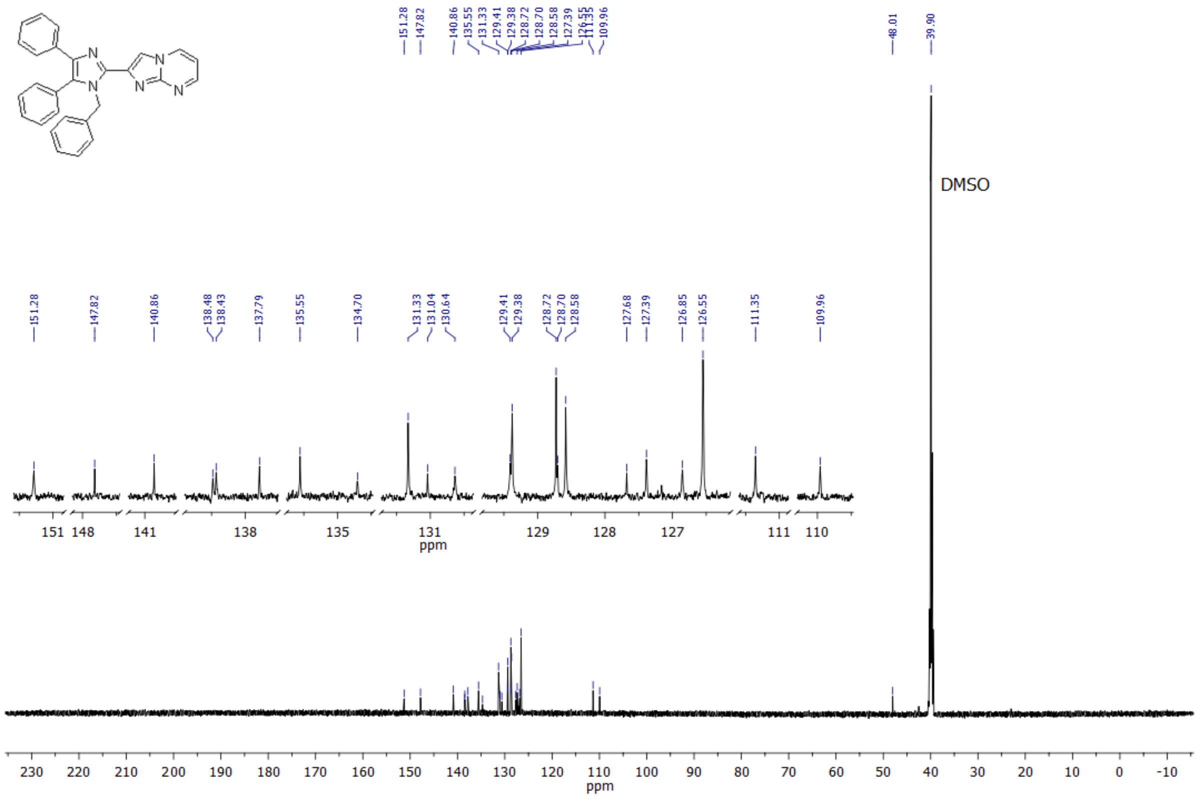

Figure S29. ^{13}C NMR spectra of compound **7**.

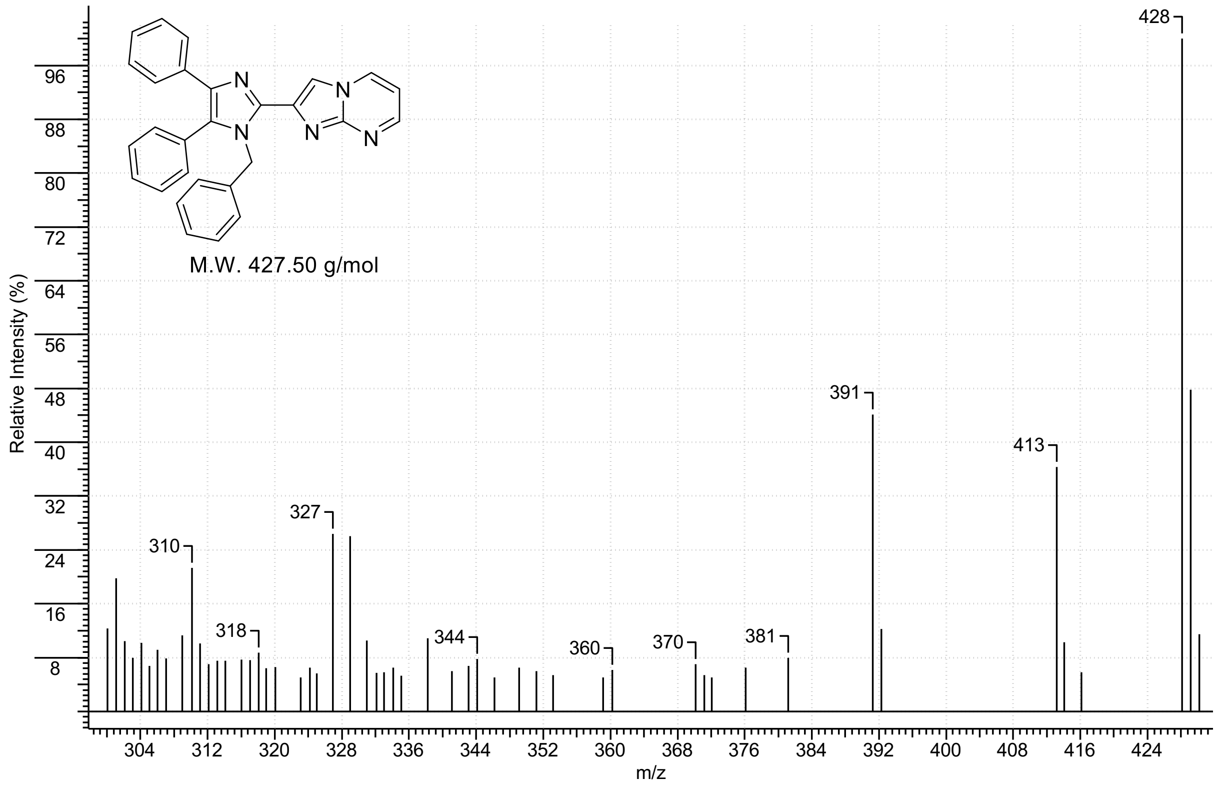

Figure S30. MS spectra of compound **7**.

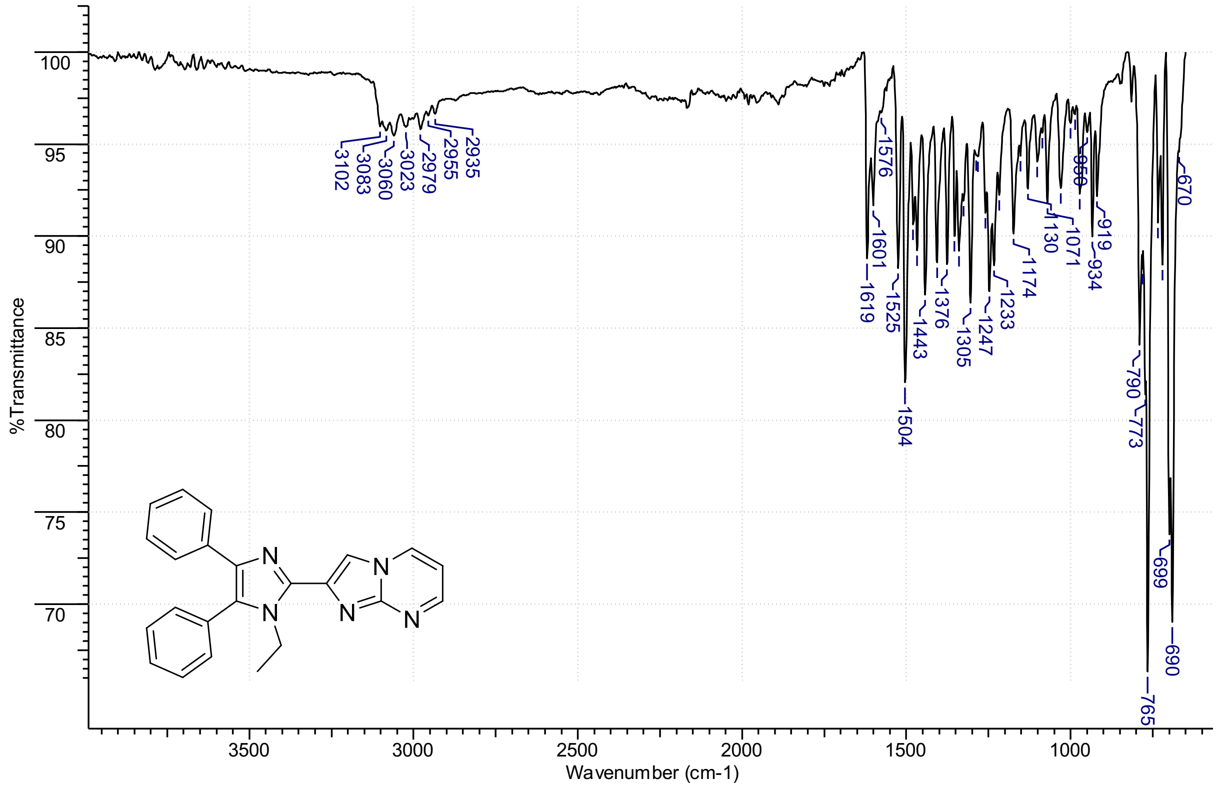

Figure S31. FT-IR spectra of compound 8.

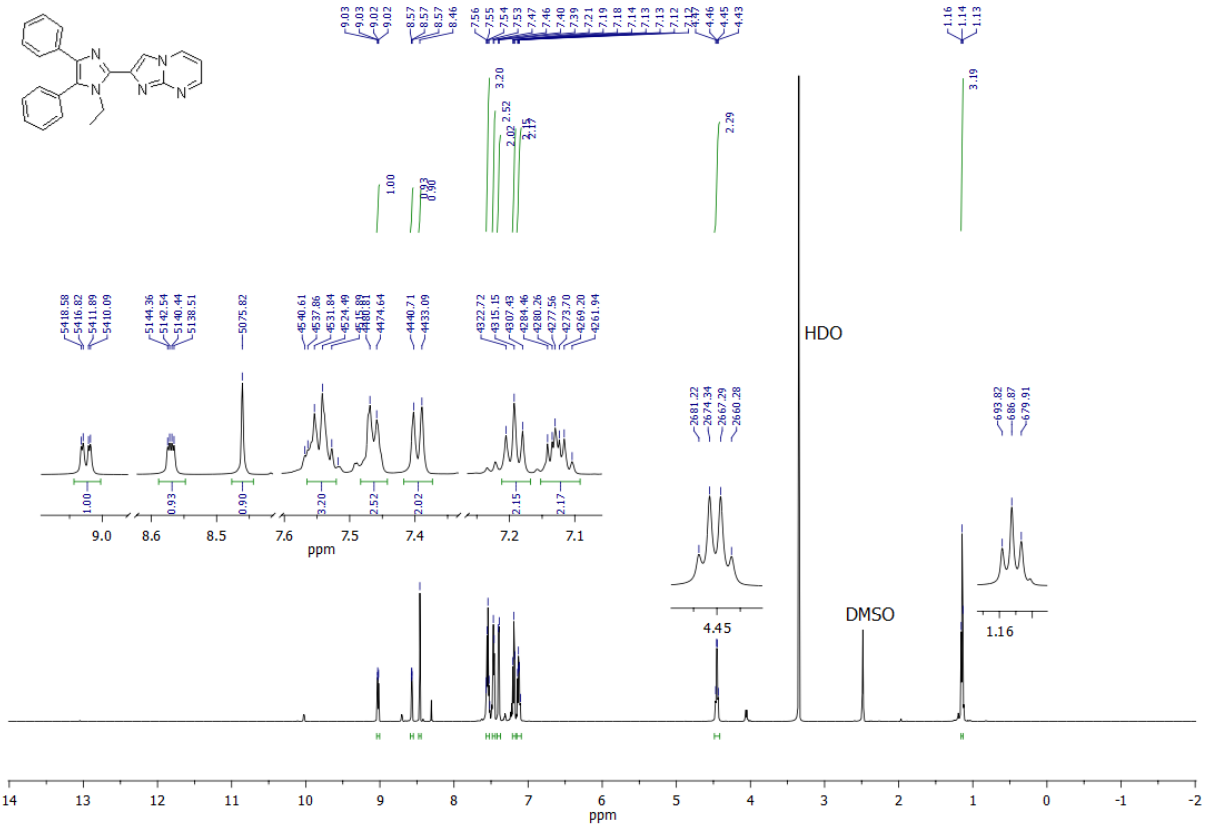

Figure S32. ^{1}H NMR spectra of compound 8.

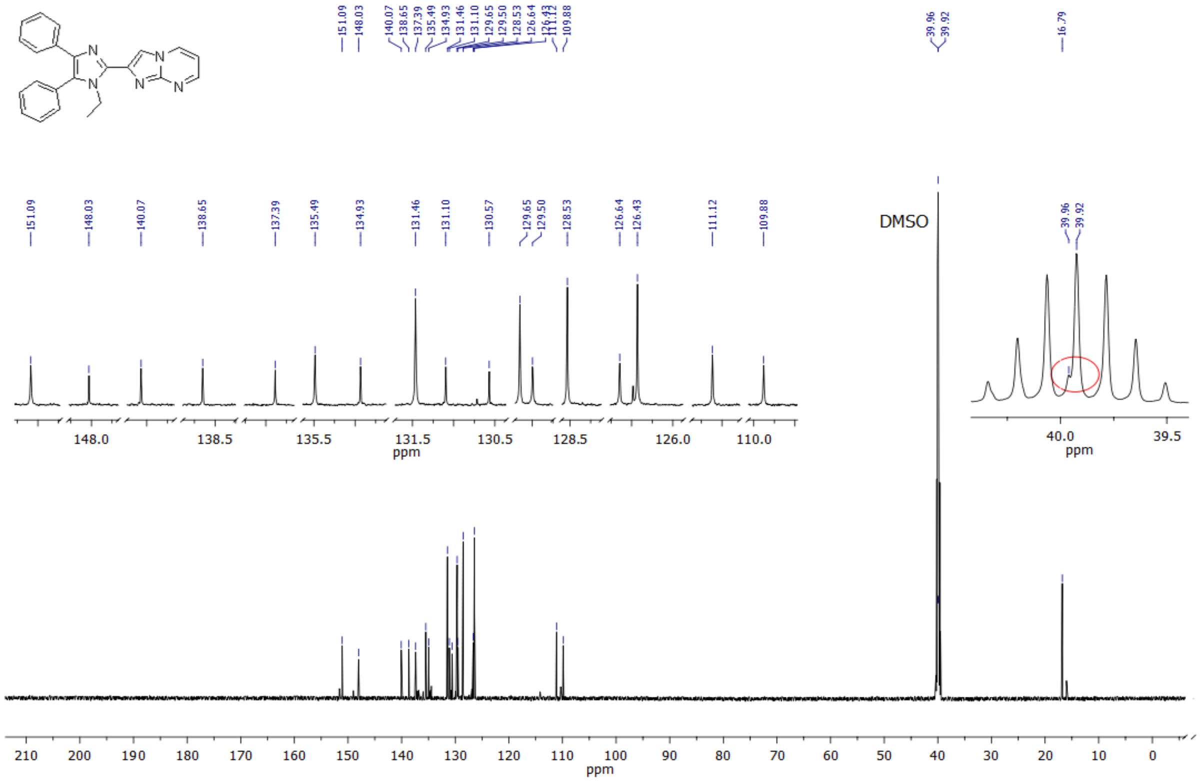

Figure S33. ^{13}C NMR spectra of compound 8.

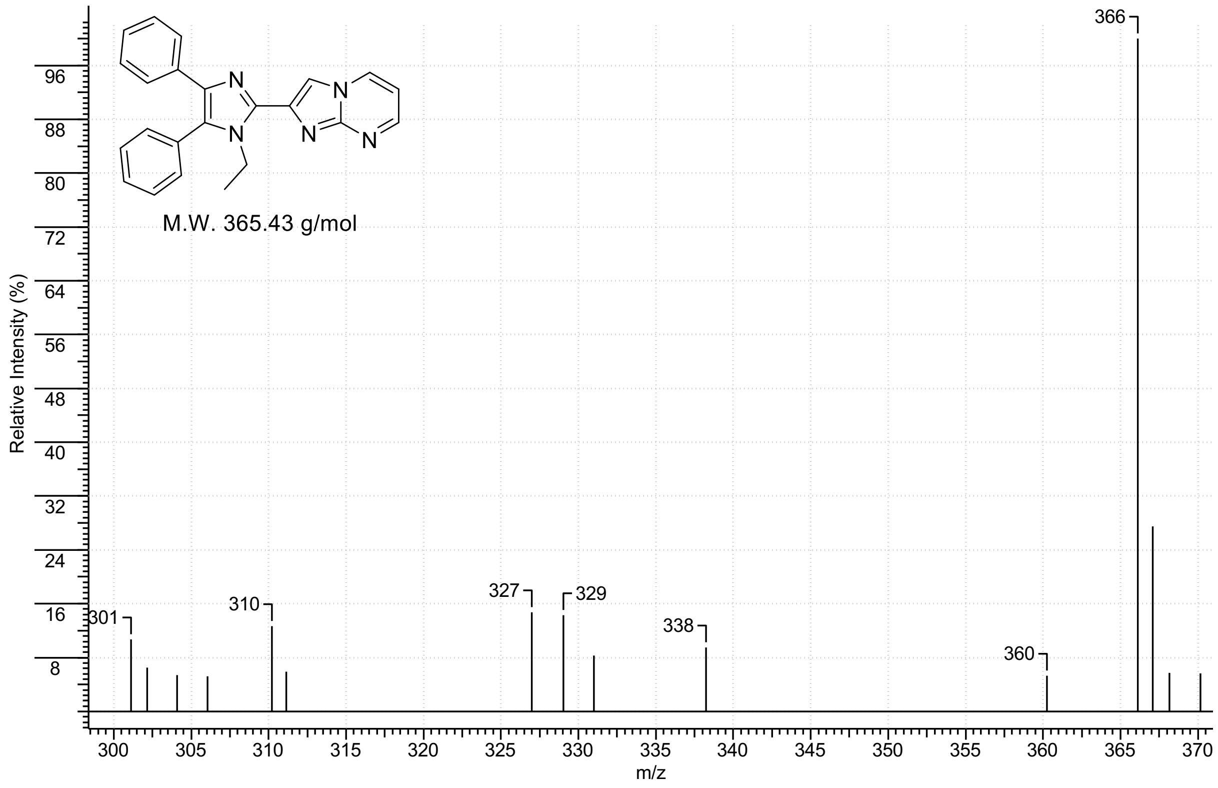

Figure S34. MS spectra of compound 8.

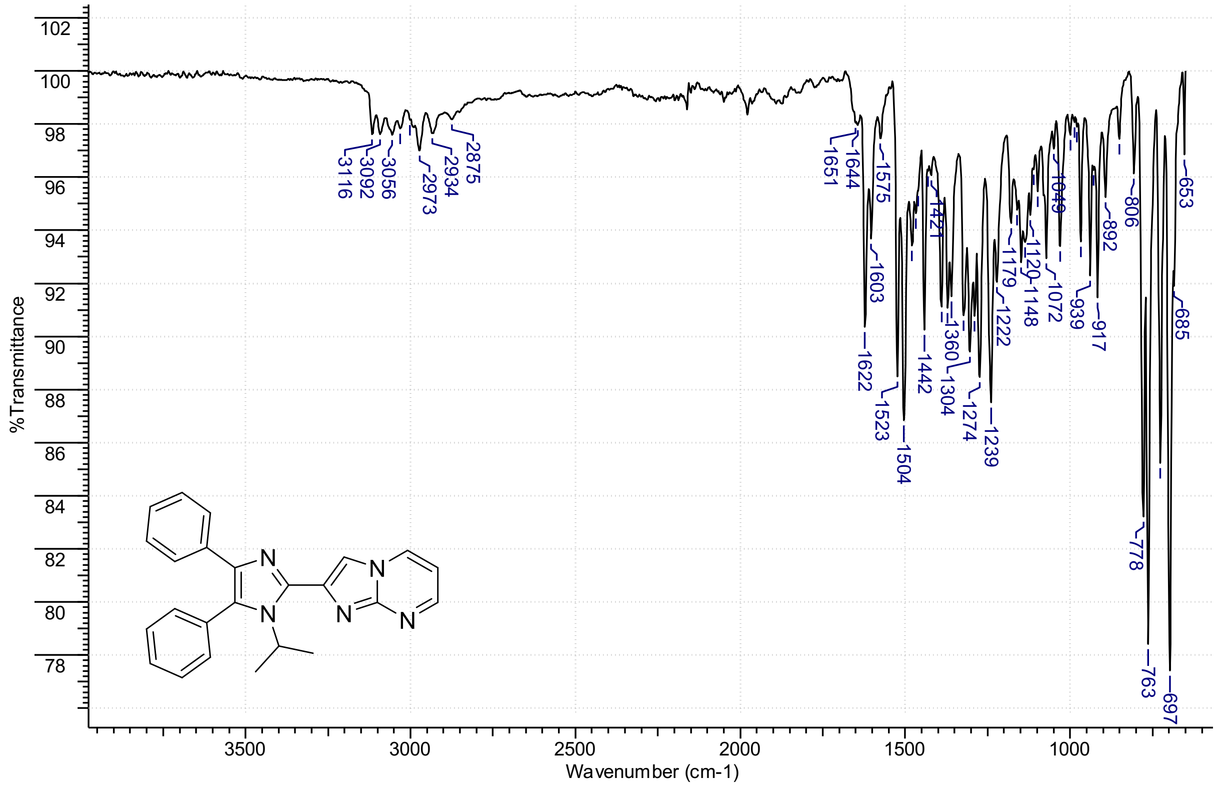

Figure S35. FT-IR spectra of compound 9.

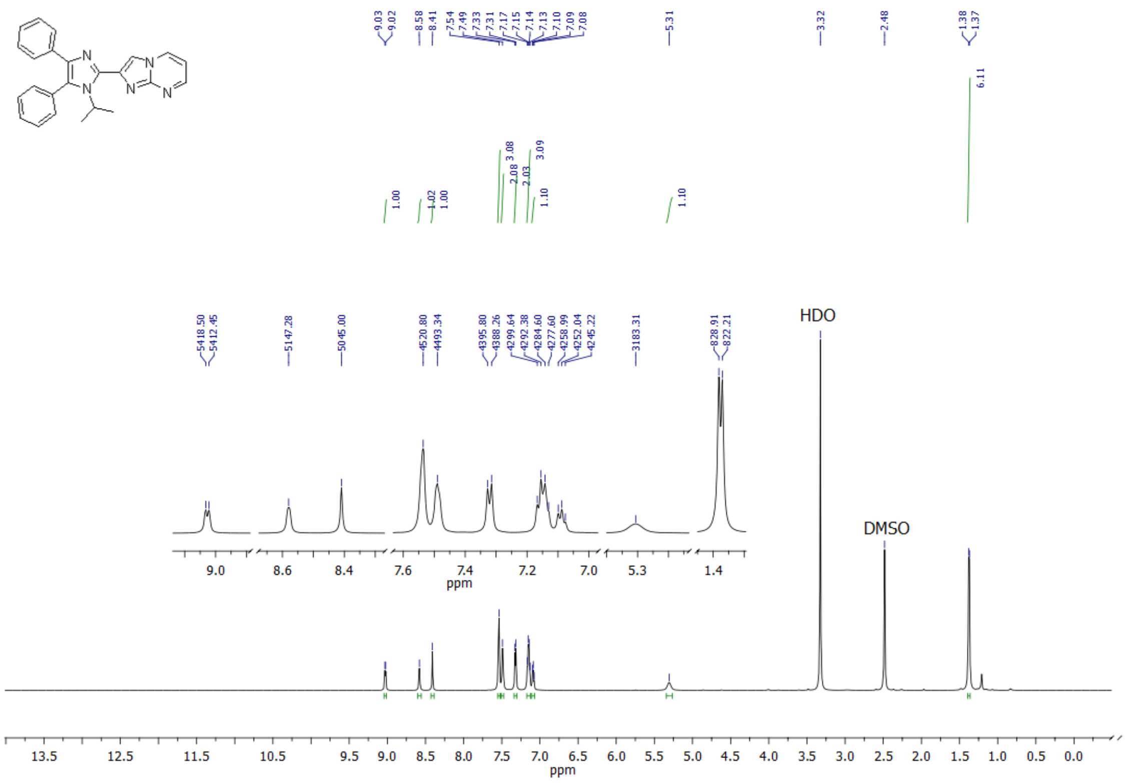

Figure S36. ^{1}H NMR spectra of compound 9.

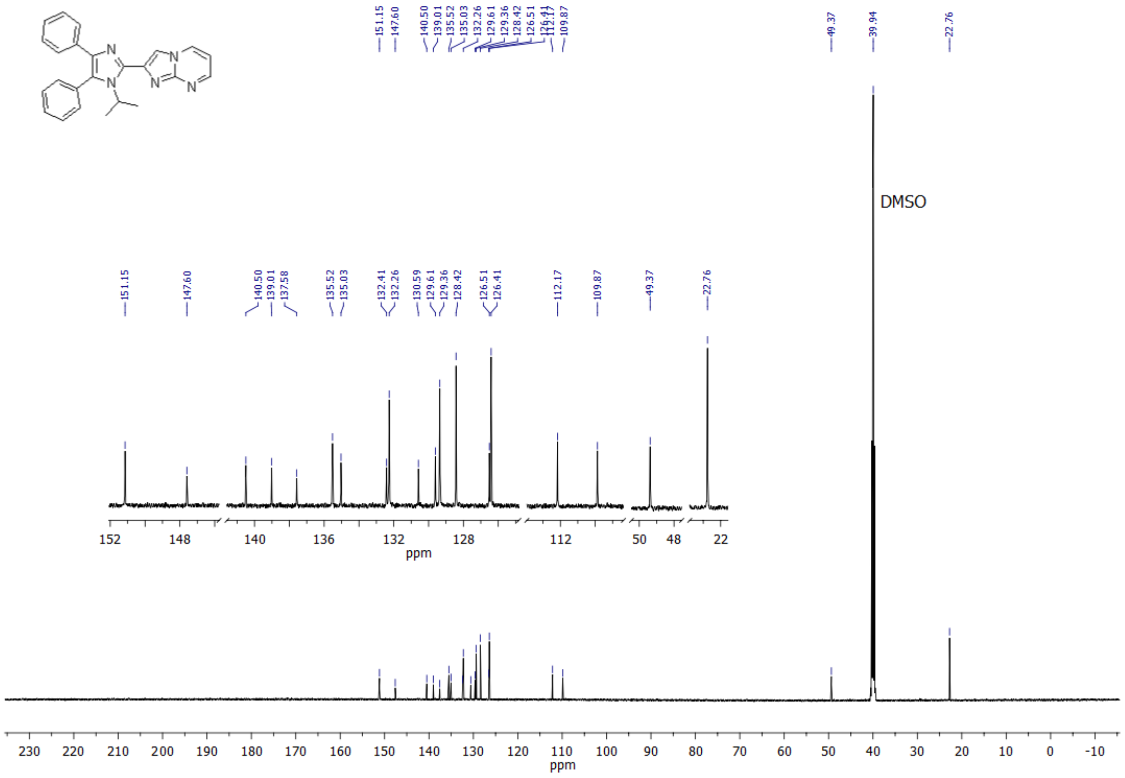

Figure S37. ^{13}C NMR spectra of compound 9.

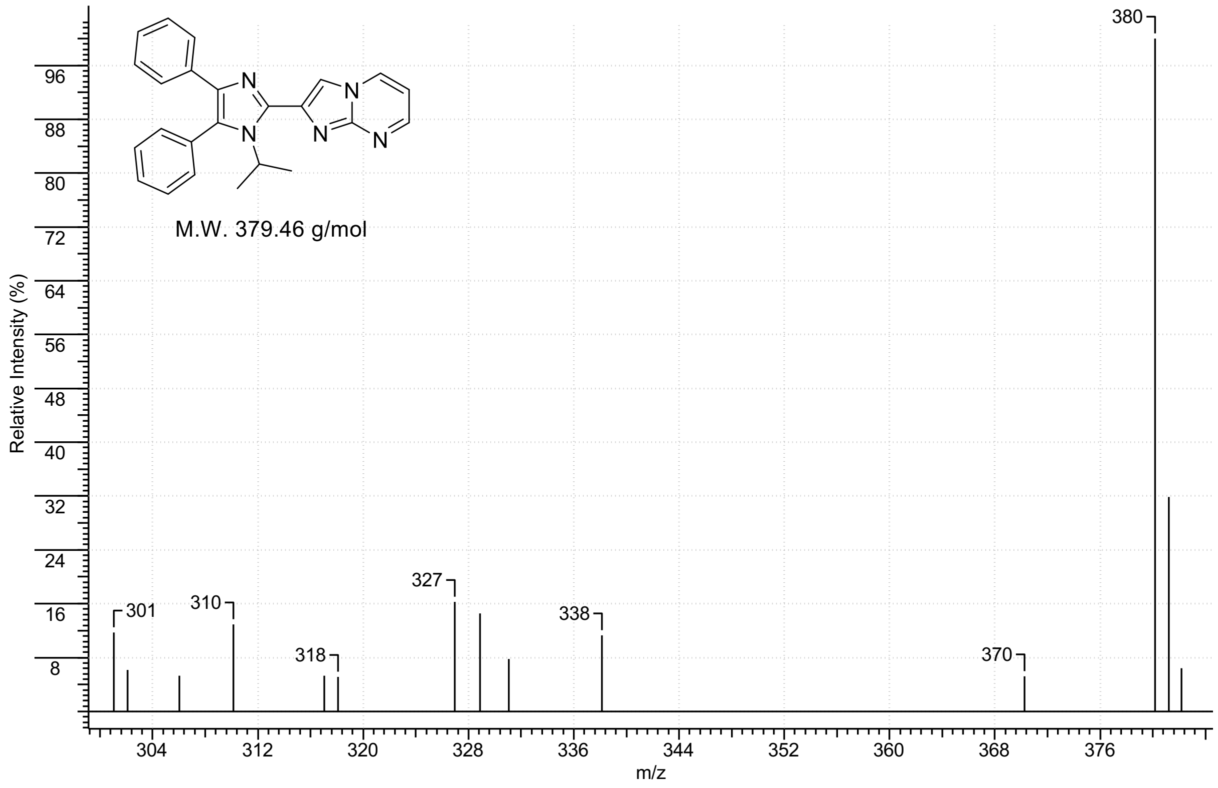

Figure S38. MS spectra of compound 9.

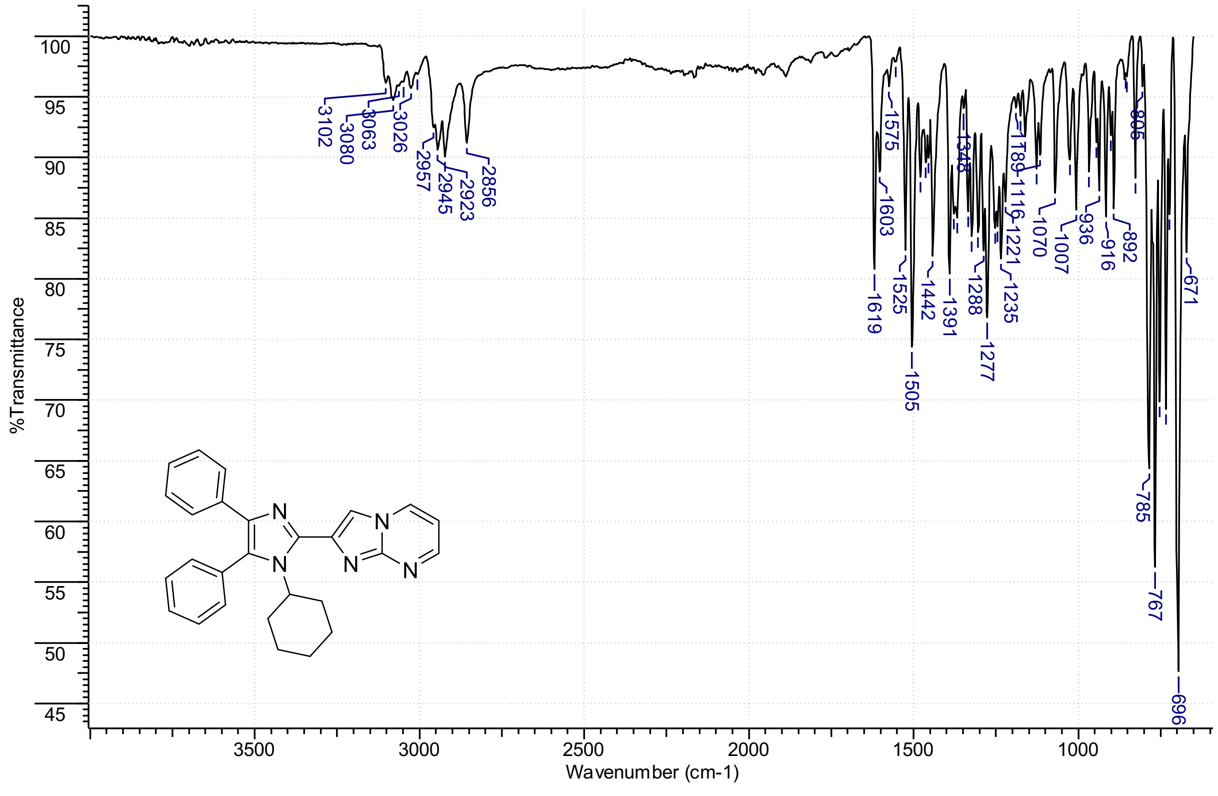

Figure S39. FT-IR spectra of compound 10.

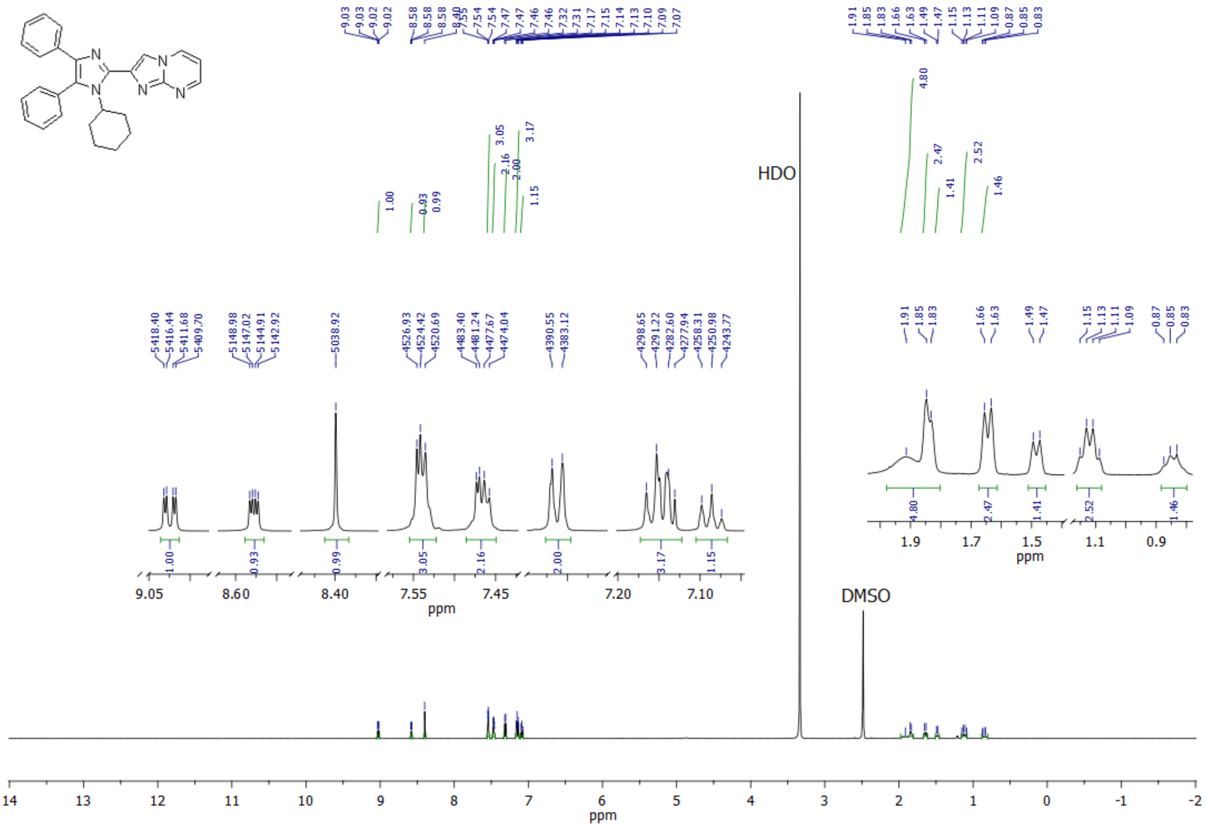

Figure S40. ^{1}H NMR spectra of compound 10.

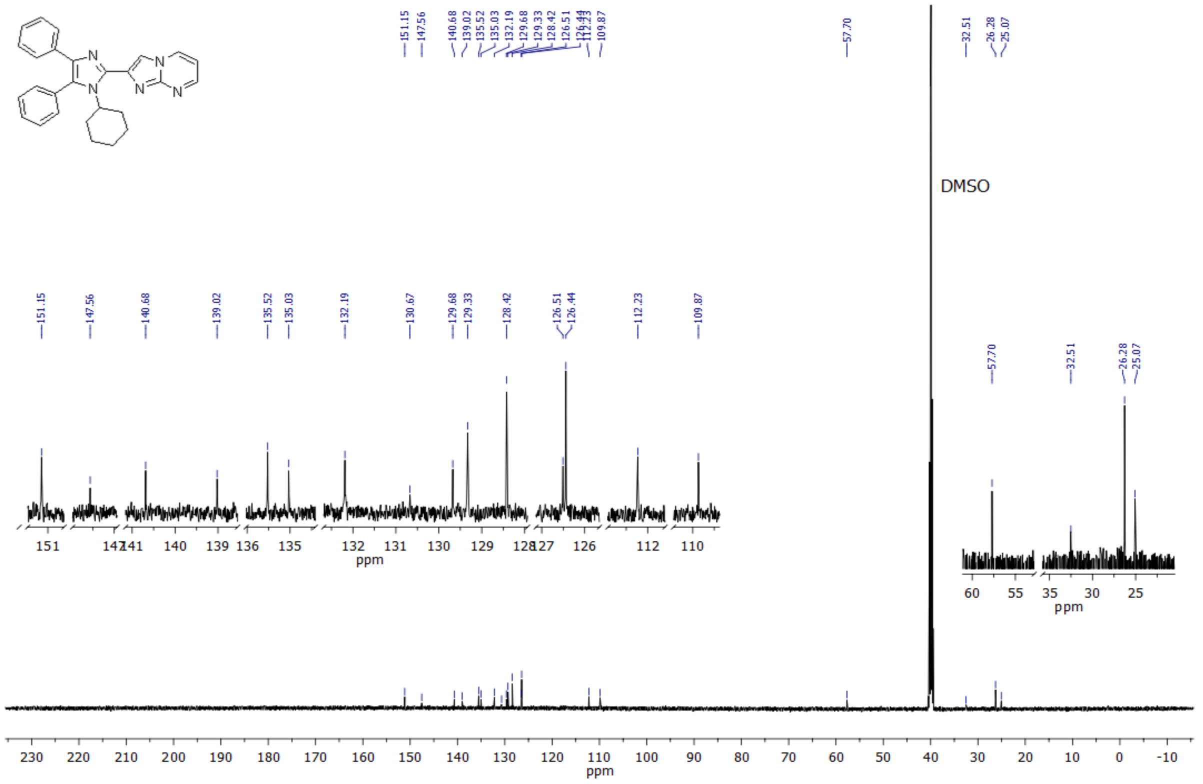

Figure S41. ^{13}C NMR spectra of compound 10.

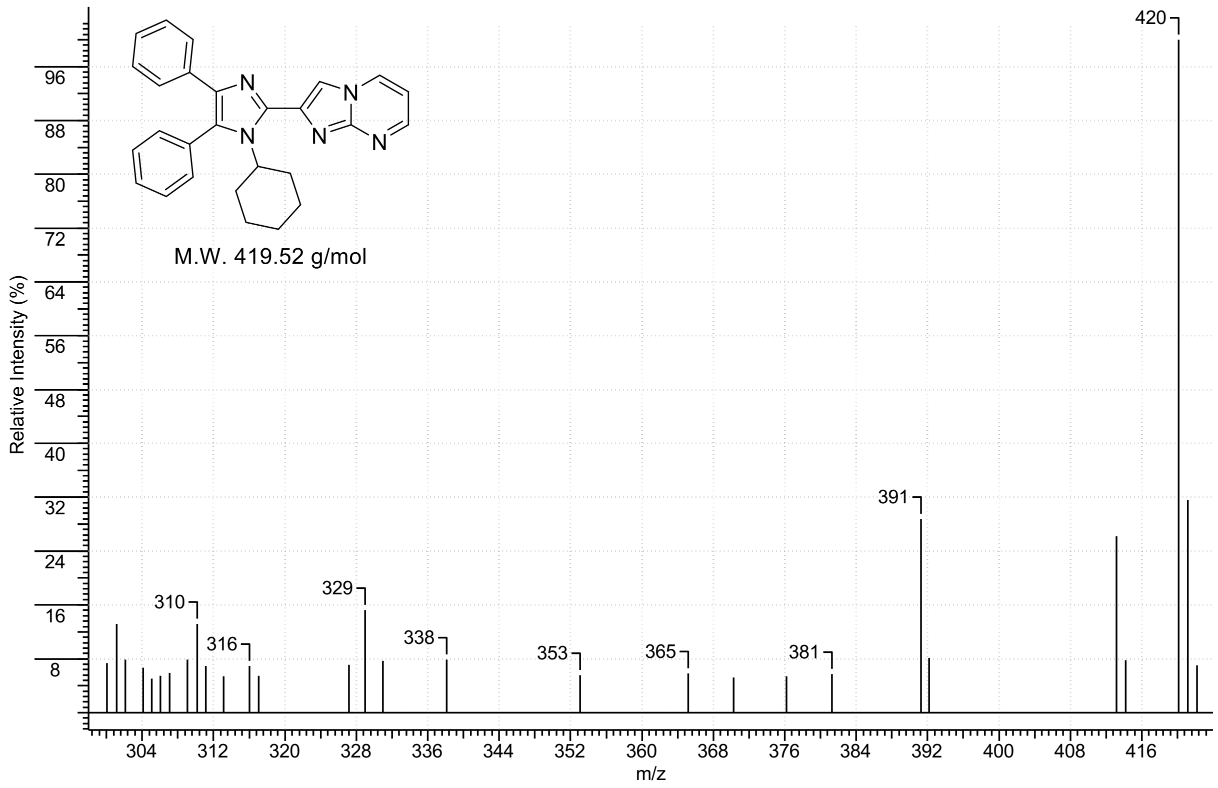

Figure S42. MS spectra of compound 10.
